# The protein composition of human adenovirus replication compartments

**DOI:** 10.1128/mbio.02144-24

**Published:** 2024-11-29

**Authors:** Paloma Hidalgo, Amada Torres, Pierre M. Jean Beltran, Gamaliel López-Leal, Luca D. Bertzbach, Thomas Dobner, S. J. Flint, Ileana M. Cristea, Ramón A. González

**Affiliations:** 1 Centro de Investigación en Dinámica Celular, Instituto de Investigación en Ciencias Básicas y Aplicadas, Universidad Autónoma del Estado de Morelos, Cuernavaca, Mexico; 2 Department of Viral Transformation, Leibniz Institute of Virology (LIV) 28367 , Hamburg, Germany; 3 Department of Molecular Biology, Princeton University, Princeton, New Jersey, USA; Centro Nacional de Biotecnologia, Madrid, Spain

**Keywords:** human adenovirus (HAdV), virus-induced compartments, viral replication, viral replication compartment (RC), histone chaperones, HMGB1

## Abstract

Human adenoviruses are double-stranded DNA viruses that replicate in the cell nucleus and induce the formation of replication compartments (RCs) that are critical in viral replication and control of virus-host interactions. RCs are specialized virus-induced subnuclear microenvironments where not only viral genome replication and expression are orchestrated but also host proteins that restrict viral replication are co-opted and subverted. The protein composition of these RCs remains largely unexplored. In this study, we isolated adenovirus RC-enriched fractions from infected cells at different times post-infection and employed a tandem mass tag-based quantitative mass spectrometry approach to identify proteins associated with RCs (data available via ProteomeXchange identifier PXD051745). These findings reveal an elaborate network of host and viral proteins potentially relevant for RC formation and function. To validate the RC-protein components identified by mass spectrometry, we employed immunofluorescence and immunoblotting techniques. Proteins previously described to colocalize in RCs in infected cells were identified in the isolated subnuclear fractions. In addition, we validated newly identified proteins associated with RCs, including the high mobility group box 1 (HMGB1), the SET nuclear proto-oncogene, the structure-specific recognition protein 1 (SSRP1), the CCCTC-binding protein (CTCF), and sirtuin 6 (SIRT6). We identified HMGB1 as a protein that binds to the viral DNA binding protein (DBP). Using shRNA knockdowns and inhibitors, we demonstrated that HMGB1 acts as a proviral factor, promoting efficient viral DNA synthesis and progeny production. Our data further suggest potential candidate targets for therapeutic intervention and provide mechanistic insights into the molecular basis of virus-host interactions.

**IMPORTANCE:**

Human adenoviruses serve as models for studying respiratory viruses and have provided critical insights into viral genome replication and gene expression, as well as the control of virus-host interactions. These processes are coordinated within virus-induced subnuclear microenvironments known as RCs. We conducted quantitative proteome analyses of RC-enriched subnuclear fractions at different times post-infection with human adenovirus species C type 5, revealing a multifaceted network of proteins that participate in the regulation of gene expression, DNA damage response, RNA metabolism, innate immunity, and other cellular antiviral defense mechanisms. Furthermore, we validated the localization of several host proteins to viral RCs using immunofluorescence microscopy and immunoblotting and identified cellular HMGB1 as a proviral factor late during infection. These findings represent the first analysis of the proteomes of isolated RCs and not only enhance our understanding of nuclear organization during infection but also shed light on the complex interplay between viral and host factors within RCs.

## INTRODUCTION

Cells partition molecular components into a variety of membrane-bound and membrane-less organelles to selectively concentrate and regulate selectively specific sets of molecules for optimal cellular metabolism and homeostasis. Infection by viruses can induce modification of the intracellular milieu and the formation of intracellular compartments necessary for efficient viral reproduction (1, 2). Although viruses have different types of genomes and replication strategies, virus-induced compartmentalization of the infected cell seems to be a common process that supports viral replication (2–7). Virus-induced intracellular compartments are generally recognized as sites of viral genome replication and transcription and therefore are commonly termed replication organelles, centers or compartments (RCs), viroplasms, or viral factories. In some cases, viruses can also induce the formation of compartments where viral progeny assemble (4, 6, 8–13).

Adenoviruses (AdVs) are non-enveloped double-stranded DNA viruses of vertebrates that, for seven decades, have proven to be excellent models for the study of various cellular processes, including DNA replication, transcription, RNA processing, cell transformation, and cell cycle regulation (14, 15). AdVs also serve as vectors for the delivery of vaccines and in gene and oncolytic therapies; approximately 70% of the global population has been vaccinated against SARS-CoV-2, with AdVs serving as prominent vaccine vectors (16–18).

Most humans have been infected by different human AdV (HAdV) types by the age of five. Clinically apparent HAdV infections usually manifest as mild upper respiratory tract infections. Unlike other respiratory viruses, HAdV outbreaks occur year-round and pose a significant risk of severe and fatal disease in the very young, the elderly, and immunocompromised patients. Currently, neither widely available vaccines nor approved anti-adenoviral drugs exist to treat HAdV infections effectively (19). Therefore, the study of HAdVs is crucial to understanding their pathogenesis and advancing viral vector development.

The genome of HAdVs is replicated and expressed in the nuclei of infected cells. The virus replication cycle is divided into an early and a late phase, delimited by the onset of viral genome replication, which occurs at 8 to 10 h post-infection (hpi) in human cell lines such as HeLa, A549, or HEK-293 (20, 21) and between 16 and 20 hpi in primary human cells, such as human foreskin fibroblasts (HFFs), respectively (22). HAdV-infected cells display a variety of mechanisms that counteract cellular antiviral responses ensuring viral replication and progeny production (23). The infection causes extensive nuclear reorganization during viral replication, which is seemingly concerted with the formation of RCs (24). Concomitant with viral early gene expression, the E2A-encoded single-stranded DNA binding protein (DBP), which has often functioned as a marker of RCs, accumulates in numerous nuclear foci (at approximately 12 and 16 hpi, depending on the cell line or cell type, respectively) that correspond to early RCs that harbor viral DNA and viral early mRNAs (24–27). The accumulation of viral early proteins in the cell nucleus leads to the initiation of viral DNA synthesis, and as viral DNA replication progresses, RCs are reshaped forming larger ring-like structures. During the late phase, viral late proteins accumulate, and progression of the viral replication cycle results in RCs coalescing into larger, morphologically more complex structures, with numerous small foci that accumulate large quantities of viral DNA, viral late mRNA and proteins, and viral particles (24, 28). Furthermore, cellular proteins that can inhibit viral replication or interfere with the viral replication cycle may be co-opted to RCs. At these sites, their inhibitory activity is subverted or repurposed to promote productive HAdV reproduction, a strategy that is also observed in cells infected by other viruses (2, 6, 24, 29–34).

HAdV-induced RCs are vital for viral replication and progeny production (35–37). Consequently, a deeper understanding of the assembly of RCs and their critical components and activities is paramount to understanding how viral replication and cellular activities are modulated. The detailed study of RCs should facilitate the elucidation of virus-host interactions, information that, in turn, could be useful for the development of effective antiviral therapies. In this work, we, therefore, set out to characterize the proteomic composition of HAdV RCs at different time points post-infection using a method that allows the isolation of RC-enriched subnuclear fractions from infected-cell nuclei (38–40), followed by a tandem mass tag-based quantitative mass spectrometry (TMT-MS) approach (41). This study unveiled a previously unreported complex network of host proteins implicated in RC-associated activities and virus-host interactions.

## RESULTS

### TMT-MS analysis of the protein composition in HAdV RCs

We have previously reported a cell-free system consisting of HAdV-RC-enriched subnuclear fractions, which contain particles with the expected morphology and allow an analysis of the regulation of viral DNA replication, transcription, and splicing (38, 40). Here, we employed this biochemical strategy to study the protein composition of HAdV-C5-induced RCs at different times post-infection.

**Fig 1:**
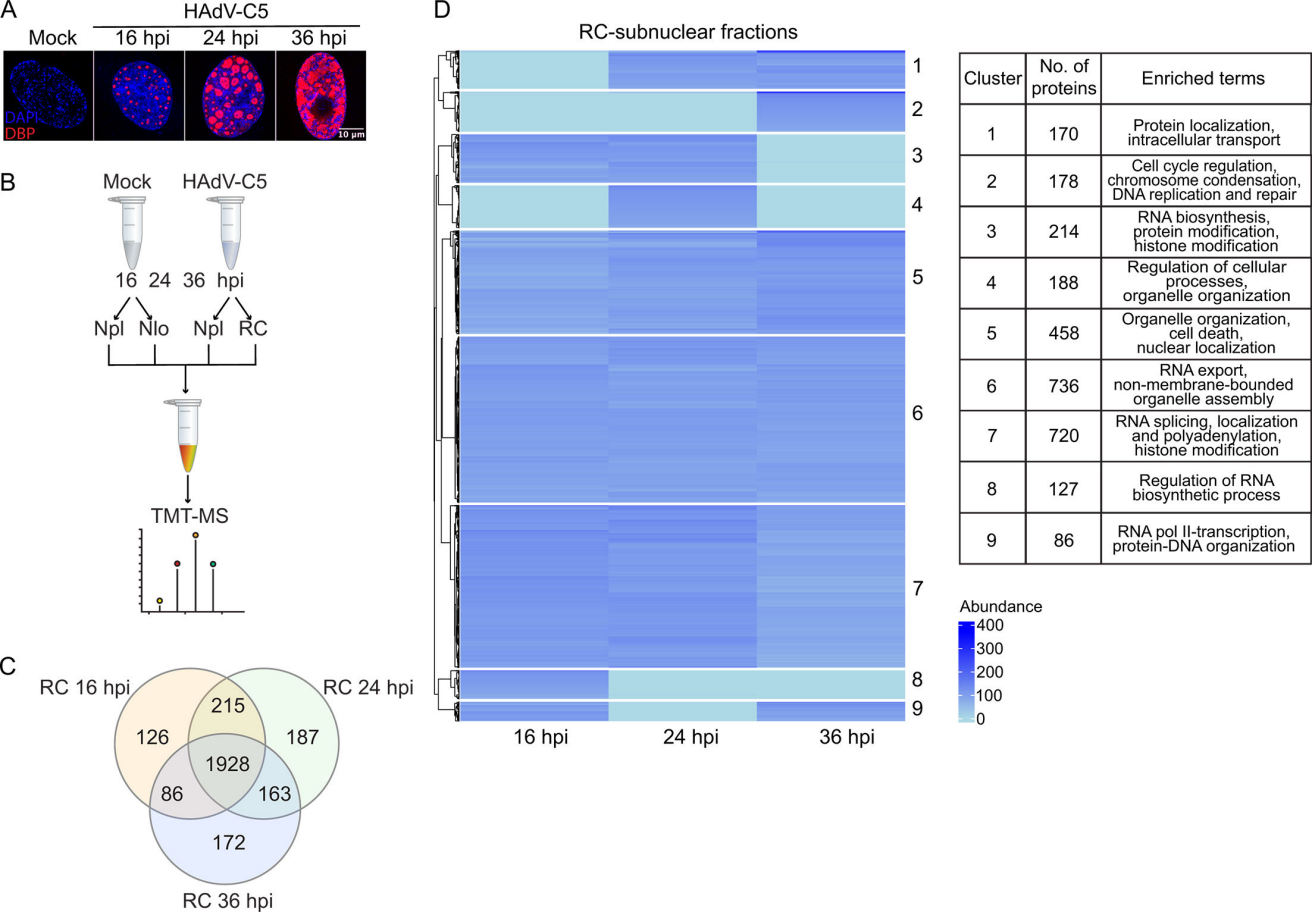
Proteins identified in RC-enriched subnuclear fractions. (**A**) HFFs infected with HAdV-C5 were fixed at 16, 24, or 36 hpi. The cells were immunolabelled to detect DBP with Alexa Fluor 555 (red) and the DNA was labeled with DAPI (blue). The samples were visualized by confocal microscopy as z-stacks, and the images are presented as maximum-intensity projections. The scale bar corresponds to 10 µm. (**B**) Diagram showing the workflow for the TMT-labeling of non-infected (mock) or HAdV-C5 infected subnuclear fractions at different times post-infection, and subsequent multiplex mass spectrometry analysis. Nlo: nucleoli; Npl: nucleoplasm. (**C**) Venn diagram showing the unique and shared number of proteins identified in RCs between the different times post-infection. (**D**) A heatmap was generated using the Euclidean distance metric for proteins identified and quantified in each RC fraction at 16, 24, and 36 hpi. Nine statistically significant clusters were determined using the gap statistic function of the Cluster package. The ontology analysis at the biological process level shows the enriched terms for each cluster, which are provided in the table.

RC-enriched fractions from HAdV-infected HFFs were isolated and analyzed by TMT-MS (42) to identify the RC-associated proteome. The time points post-infection selected represent an early time post-infection (16 hpi), when E2-DBP early RCs are readily detected as small, spherical, nuclear foci; an intermediate time point (24 hpi), when viral DNA replication reaches the maximum rate of synthesis and RCs display a combination of spherical and ring-like morphologies; and a late time point (36 hpi), when RCs coalesce and occupy a large proportion of the cell nucleus (22, 24, 36, 43). RCs range in diameter from 0.3 to 2 µm (44, 45). We first confirmed that these isolated particles match the size of RCs within infected cells by dynamic light scattering (DLS; Fig. S1). Representatives of known morphology of RCs at different times post-infection, imaged through immunofluorescence (IF) confocal microscopy with the E2A-DBP protein, and the experimental MS-strategy used for these studies are shown in Fig. 1A and B, respectively.

A total of 4,412 proteins, including 26 adenoviral proteins, were identified in all combined samples and conditions, which consisted of the RC-fractions and the surrounding nucleoplasm fractions (Npl), obtained from mock-infected and HAdV-C5-infected cell nuclei harvested at the three times post-infection as described above (Table S1). The proteins identified were consistent across three biological replicates, and the average Pearson correlation coefficient (PCC), R, was higher than 0.9, indicating that the data were reproducible and reliable.

To define the proteins that were associated with RCs at different times post-infection, the data obtained by MS were analyzed via several strategies, first by analysis of the proteins identified in the RC-proteome (Fig. 1; Table S2). A total of 2,877 proteins were identified and quantified in the samples from the three times post-infection. Clustering analysis resulted in nine groups, as indicated in the heatmap of Fig. 1D, showing that proteins that participate in chromatin remodeling, DNA replication and repair, and RNA biogenesis were associated with RC fractions.

**Fig 2:**
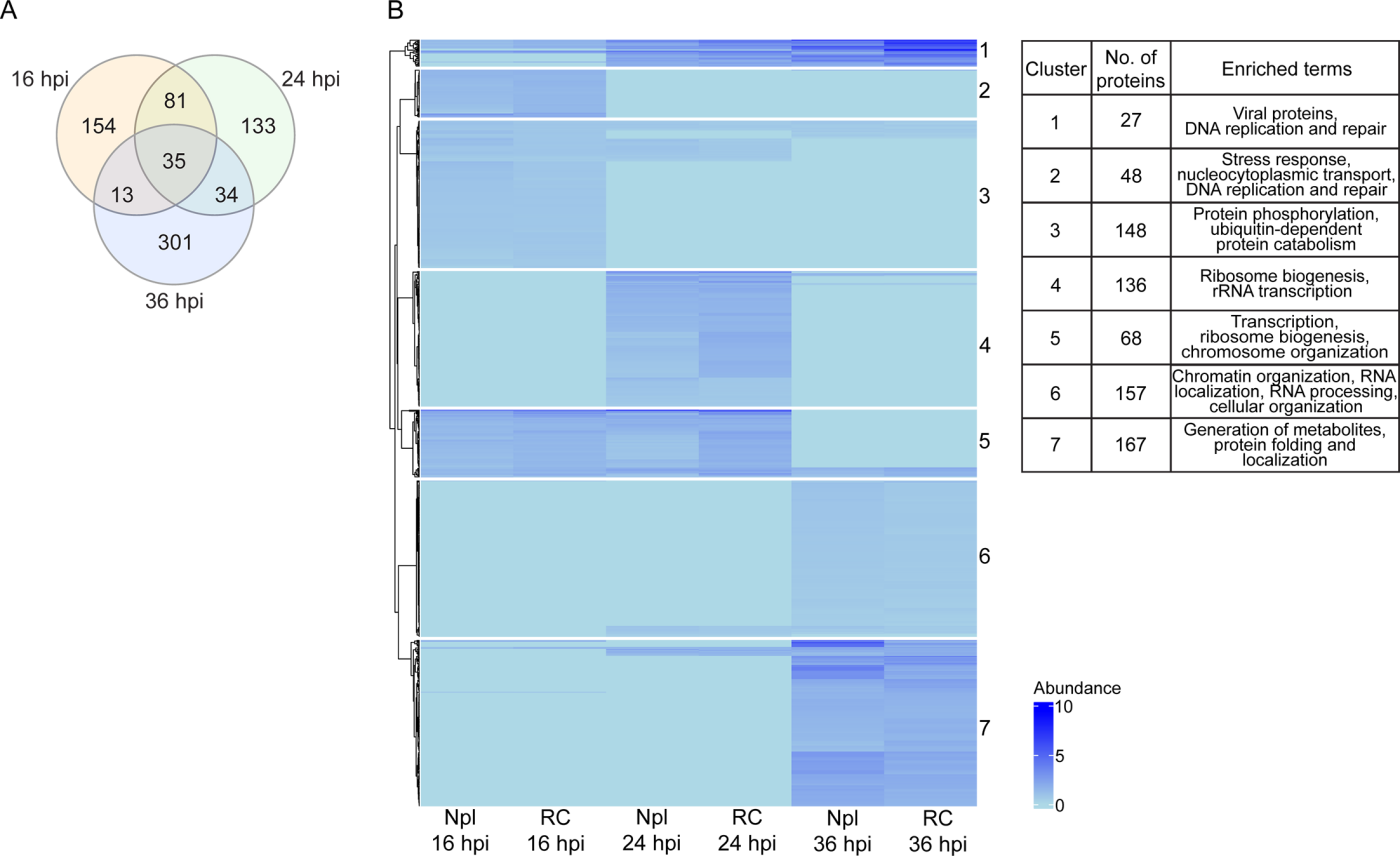
Temporal TMT-MS analysis of subnuclear fractions (multivariate analysis). (**A**) Venn diagram showing the unique and shared number of proteins between the different times post-infection. (**B**) A heatmap was generated using the Euclidean distance metric for proteins identified and quantified in each HAdV-C5 subnuclear fraction at 16, 24, and 36 hpi (in comparison to mock-infected cells). Only proteins without missing values are shown (751 proteins). Seven statistically significant clusters were determined using the *gap* statistic function of the cluster package. The enriched biological process terms for each cluster are provided in the table.

A multivariate analysis was performed to focus on the proteome of the cell nucleus and the alterations induced by HAdV infection. For mock-infected cells, the RC fractions correspond to nucleolus (Nlo)-enriched fractions, as they are also composed of proteins and nucleic acids and share similar dimensions with RCs (38). Therefore, the total proteome of the HAdV-infected cell nucleus (RC and Npl fractions) was compared with the proteome of mock-infected cells (Nlo and Npl) to identify enriched proteins in the RC fractions of HAdV-infected cells at the three time points post-infection (Fig. 2). A total of 751 proteins (including 22 viral proteins; Fig. 2B, clusters 1 and 2, and Table S3) passed the quality control (as described in the Materials and Methods). The numbers of enriched proteins at each time point were 283 at 16 hpi, 283 at 24 hpi, and 383 at 36 hpi, with 154 proteins exclusive for 16 hpi, 133 for 24 hpi, and 301 for 36 hpi, and 35 proteins enriched in all three time points (Fig. 2A). Cluster analysis produced seven protein groups and showed that nucleoplasm and RC display temporally similar composition, albeit with clear differences in abundance (Fig. 2B; Table S3). Viral proteins, which grouped in cluster one with chromatin binding proteins, such as the high mobility group B1 (HMGB1), proliferating cell nuclear antigen (PCNA), and ribonucleotide reductase catalytic subunit M1 (RRM1), progressively increased from 16 hpi to 36 hpi (Tables S1 and S3). In contrast, most proteins in cluster 3 (protein phosphorylation, ubiquitin-dependent protein catabolism) and all proteins in cluster 2 (stress response, nucleocytoplasmic transport, DNA synthesis and repair) decreased after 16 hpi. Most proteins in cluster 6 (chromatin organization, RNA localization, RNA processing, cellular organization) and 7 (generation of metabolites, protein folding, and localization) were underrepresented at 16 and 24 hpi and increased by 36 hpi. Proteins in cluster 4 (ribosome biogenesis, rRNA transcription) increased transiently at 24 hpi, whereas most proteins in cluster 5 (transcription, ribosome biogenesis, chromosome organization) decreased by 36 hpi (Fig. 2B).

**Fig 3:**
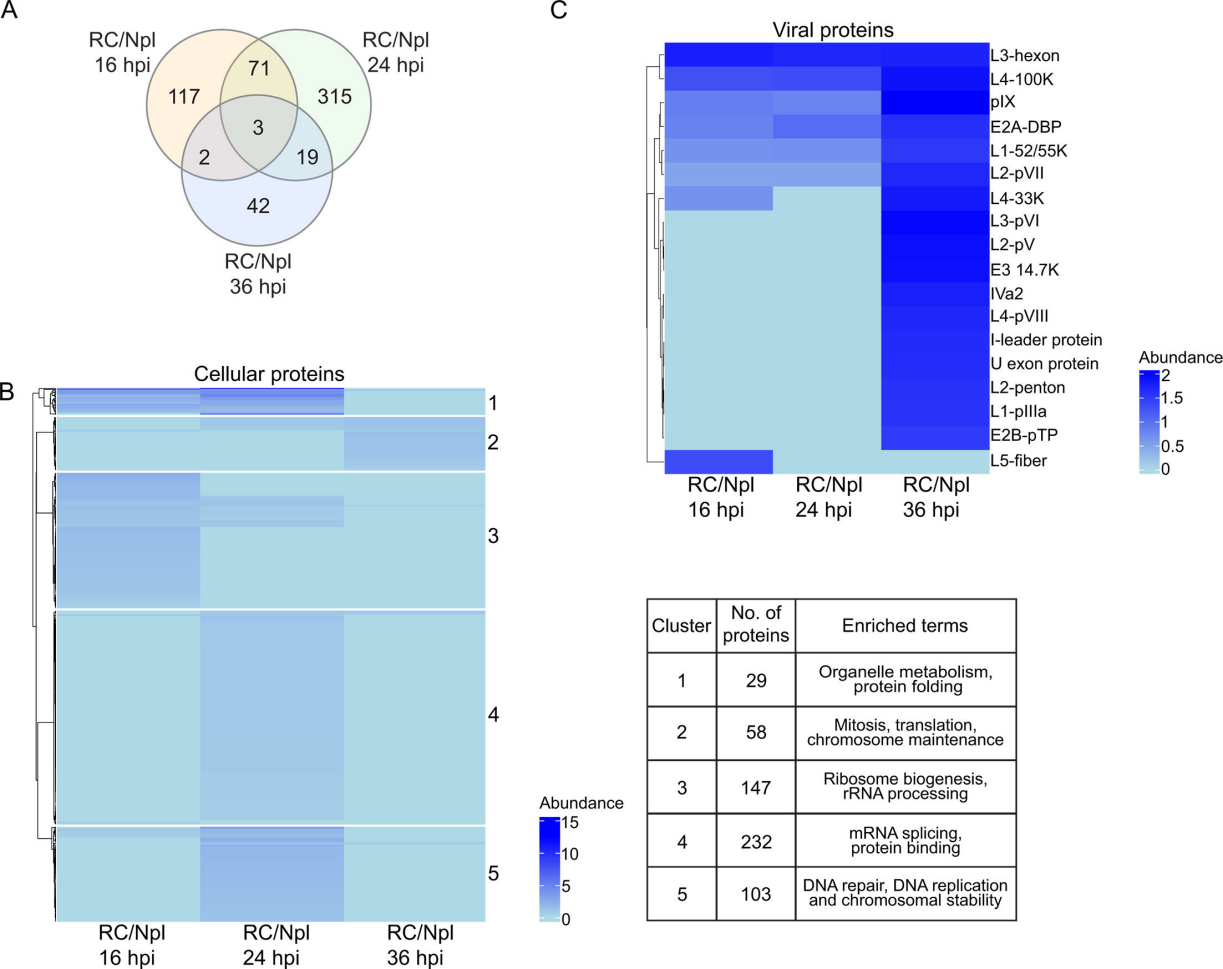
Proteins enriched in RC-subnuclear fractions (univariate analysis). To evaluate the proteins enriched in RCs relative to Npl subnuclear fractions, the ratio RC/Npl for each protein was calculated. (**A**) Venn diagram of the RC/Npl ratio between 16, 24, and 36 hpi. (**B**) A heatmap was generated using the Euclidean distance metric for the RC/Npl ratio of cellular proteins. Five statistically significant clusters were determined. The enriched biological process terms for each cluster are provided in the table. (**C**) Hierarchical heatmap of the RC/Npl ratio of viral proteins.

We then identified differential proteins between RC and nucleoplasm (Npl) fractions at each of the three time points, 16, 24, and 36 hpi (Fig. 3). A total of 569 cellular proteins were significantly enriched in this analysis in at least one time point. Similar to the distribution obtained with the multivariate analysis (Fig. 2A), the number of RC-enriched proteins exclusive to each individual time point was larger than the number of proteins shared between the different time points (Fig. 3A), indicating that the composition of RCs and the protein exchange with the surrounding nucleoplasm is highly dynamic.

When we examined cellular proteins (Fig. 3B; Table S4) in terms of their enrichment in RCs, we observed the highest abundance of proteins predominantly localized in RCs at 24 hpi compared with the Npl. The proteins in this heatmap fell into five clusters, with terms that included mRNA splicing, ribosome biogenesis, DNA repair and replication, and chromosome maintenance. The viral proteins (Fig. 3C), which consisted of 18 proteins, were highly enriched in RCs compared with the Npl at 36 hpi. As expected, DBP was enriched in RCs and increased with the progression of the viral replication cycle, whereas most other viral proteins showed a similar distribution between RCs and Npl (Fig. 2B). These results indicate that there is a selection of the viral and cellular proteins that are specifically enriched in RCs.

**Fig 4:**
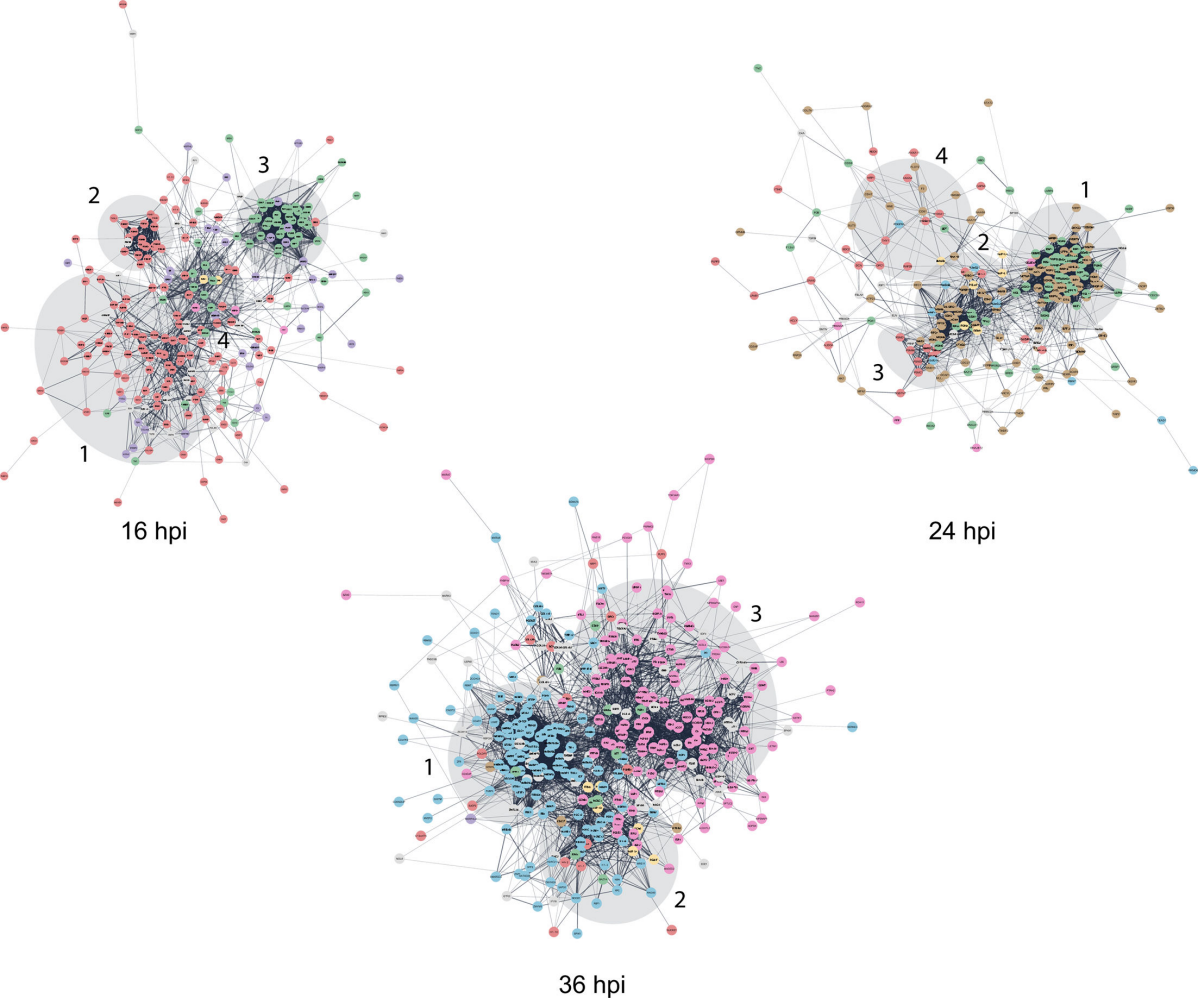
Physical and functional interactions between RC proteins. STRING network visualization of proteins from RC-enriched subnuclear fractions was performed to identify physical and functional protein associations at the times post-infection shown. The colors represent proteins from the clusters in Fig. 2B (Table S3): yellow, cluster 1; purple, cluster 2; red, cluster 3; brown, cluster 4; green, cluster 5; blue, cluster 6; pink, cluster 7. The networks were obtained with default settings for a full STRING network, displaying edges by confidence mode with a minimum interaction score of 0.4; a maximum FDR ≤ 0.05; a minimum strength ≥0.01; a minimum count of 2, and displaying no disconnected nodes.

We then used the Search Tool for the Retrieval of Interacting Proteins database (STRING) (46) to obtain further insights into the network of physical and functional interactions between RC proteins. This analysis confirmed networks of proteins previously reported to be relevant for RC-associated activities (such as p53 (47) and MDM2 (48)) and proteins selected from the RC proteome (Fig. S2; Table S2). In addition, the analysis revealed a variety of networks that have not been described previously. The networks shown in Fig. 4 represent the proteins per time point obtained from the multivariate analysis, which correspond to the proteins enriched in HAdV-infected cells (RC and Npl fractions) compared with mock-infected cells (Nlo and Npl) at each of the three time points post-infection (Table S3).

At 16 hpi, 283 proteins formed a large network with four distinguishable sub-networks (Fig. 4). One sub-network (formed mostly by proteins from cluster 3, identified with the terms: protein phosphorylation, ubiquitin-dependent protein catabolism in Fig. 2B and shown in red) included biological processes, such as cell organization, cell proliferation, cell division, cell death, and metabolic processes, among others (Table S3). A second well-defined sub-network also formed by proteins from cluster 3 was enriched with the proteasome core complex associated with the regulation of virus-host interactions (PSMA3, PSMA4, PSMA7, PSMB1, PSMB5, PSMB6, and PSMB7). A third sub-network (formed mostly by proteins from cluster 5 and some from cluster 2 in Fig. 2B, shown in green and purple, respectively), included proteins identified with the terms: transcription, ribosome biogenesis, chromosome organization; and stress response, nucleo-cytoplasmic transport, and DNA synthesis and repair, respectively. The fourth sub-network (formed by proteins from clusters 1, 3, and 5 in Fig. 2B, shown in yellow, red, and green, respectively) included functions related to DNA repair and replication, grouped by three proteins, TOP2A, CDK1, and PCNA with a high number of interactions. Proliferating cell nuclear antigen (PCNA) connected the four networks and is a protein known to localize in RCs in infected cells (49).

The node created by PCNA at 16 hpi (Fig. S2; Table S2) includes known DBP binding partners (USP7), proteins that colocalize with DBP in RCs (RPA2, PCNA), chromatin remodelers or chaperones HMGB 1–3 proteins, FACT (SSRP1 and SUPT16H), SET, HDAC1, and CTCF, the latter regulates adenovirus DNA replication and gene expression and therefore is expected to be in RCs (24, 36, 49–53).

At 24 hpi, the functionally relevant protein interactions were subdivided into four sub-networks (Fig. 4). One sub-network (formed by proteins from clusters 4 and 5 in Fig. 2B, shown in brown and green, respectively) consisted mainly of proteins associated with the metabolism of RNA. A second sub-network (formed by proteins from clusters 4, 5, 6, and 1 in Fig. 2B, shown in brown, green, blue, and yellow, respectively) comprised proteins related to DNA replication and repair and was enriched in proteins from the minichromosome maintenance complex (MCM2-7). A closely connected third sub-network (formed by proteins from clusters 3, 4, and 6 in Fig. 2B, shown in red, brown, and blue, respectively) consisted of proteasome-related proteins. A fourth sub-network was mainly formed by proteins from clusters 3 (red) and 4 (brown) from Fig. 2B. Interestingly, this cluster also contained proteins related to chromatin regulation and histone modification, like HMGB1, HMGB3, SET, NAA10, and NAA15. The adenoviral protein VII interacts with HMGB1 and SET, regulating cellular and viral chromatin (reviewed in (54)), as discussed below. Focusing on proteins of interest at 24 hpi, a network with p53 (TP53) at the central node that included several proteins also present at 16 hpi was observed (Fig. S2). Important metabolic pathways in this network were mismatch repair, homologous recombination, and DNA replication. HMGB1, a p53 binding partner (55), was part of this cluster.

At 36 hpi, 383 proteins formed three prominent sub-networks (Fig. 4). Two closely connected sub-networks (formed mostly by proteins from cluster 6 in Fig. 2B, shown in blue) consisted of proteins associated with RNA-binding, mRNA processing. The second sub-network (formed by proteins from clusters 6, 7, 5, 1, and 3 in Fig. 2B, shown in blue, pink, green, yellow, and red, respectively) included two closely associated groups with proteins participating in DNA damage/telomere stress-induced senescence pathways and chromatin regulator factors (56). The HMGB1 protein was part of the second sub-network, which was also closely connected to a group that included PCNA and proteins associated with homologous recombination, cell cycle regulation, and DNA repair. A large third sub-network (formed mostly by proteins from cluster 7 in Fig. 2B, shown in pink) contained proteins implicated in various activities, including the generation of metabolites, protein folding, and localization. At this time post-infection, where HMGB-proteins were represented, a central node was also created by p53 (Fig. S2; Table S2).

### Validation of RC-protein components identified by TMT-MS

**Fig 5:**
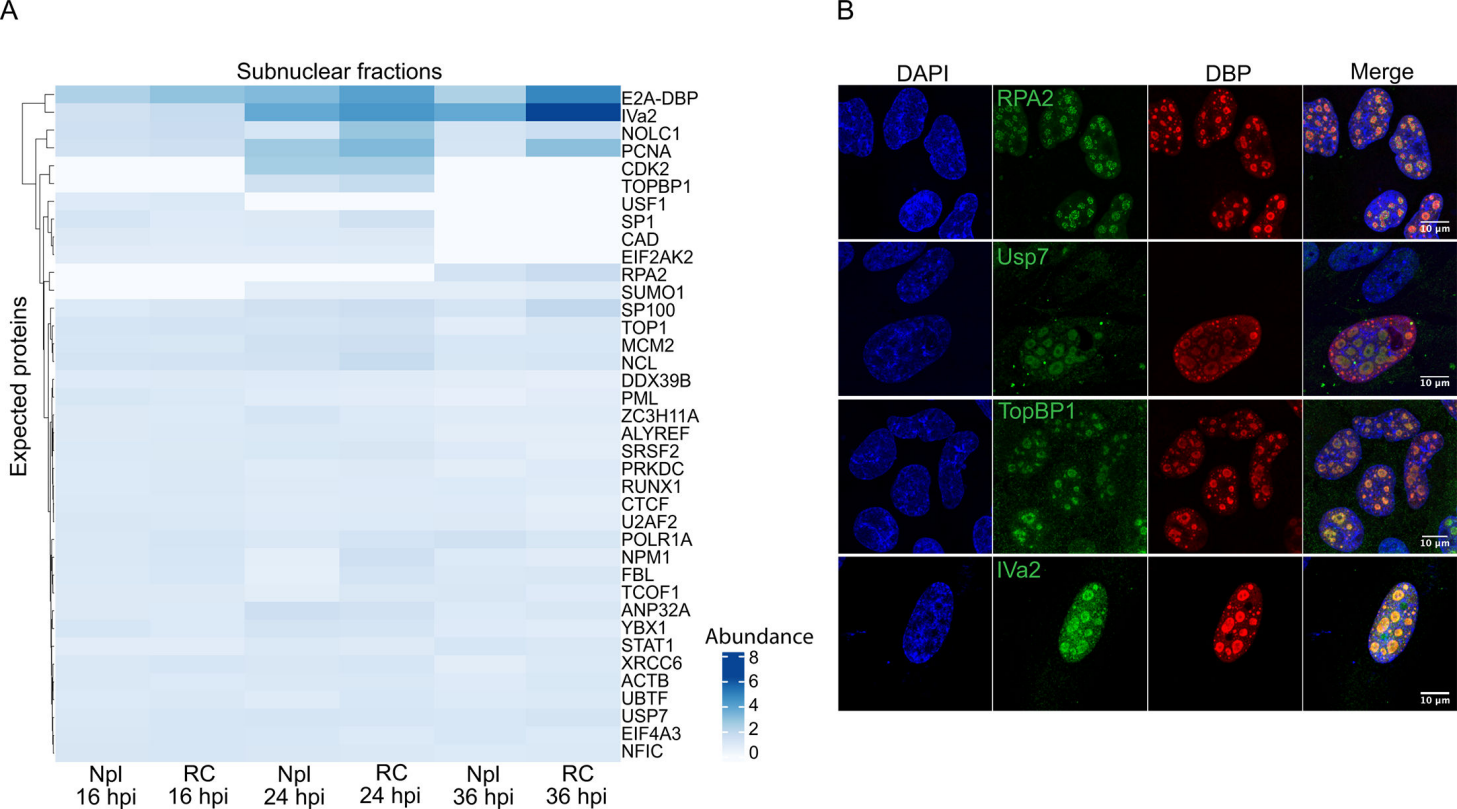
Proteins known to co-localize with DBP in infected cells are identified by TMT-MS analysis of RC-enriched subnuclear fractions. (**A**) Hierarchical heatmap of proteins expected to be in RCs and identified in the TMT-MS analysis. (**B**) HFFs infected with HAdV-C5 were fixed at 24 hpi, and RPA2, Usp7, TopBP1, and IVa2 (green) were co-stained with DBP (red) and DNA (DAPI, blue). The samples were visualized by confocal microscopy as z-stacks, and the images are presented as maximum-intensity projections. The scale bars correspond to 10 µm.

As positive controls, we initially verified that proteins known to colocalize in RCs, those that bind to DBP, and proteins required for RC-associated activities were identified in the MS analysis (Fig. 5A) (24, 36, 51, 52, 57–59). Examples of these proteins are shown in Fig. 5B in IF images of HAdV-C5-infected cells that display colocalization with DBP of RPA2, USP7, TopBP1, and IVa2 in RCs.

**Fig 6:**
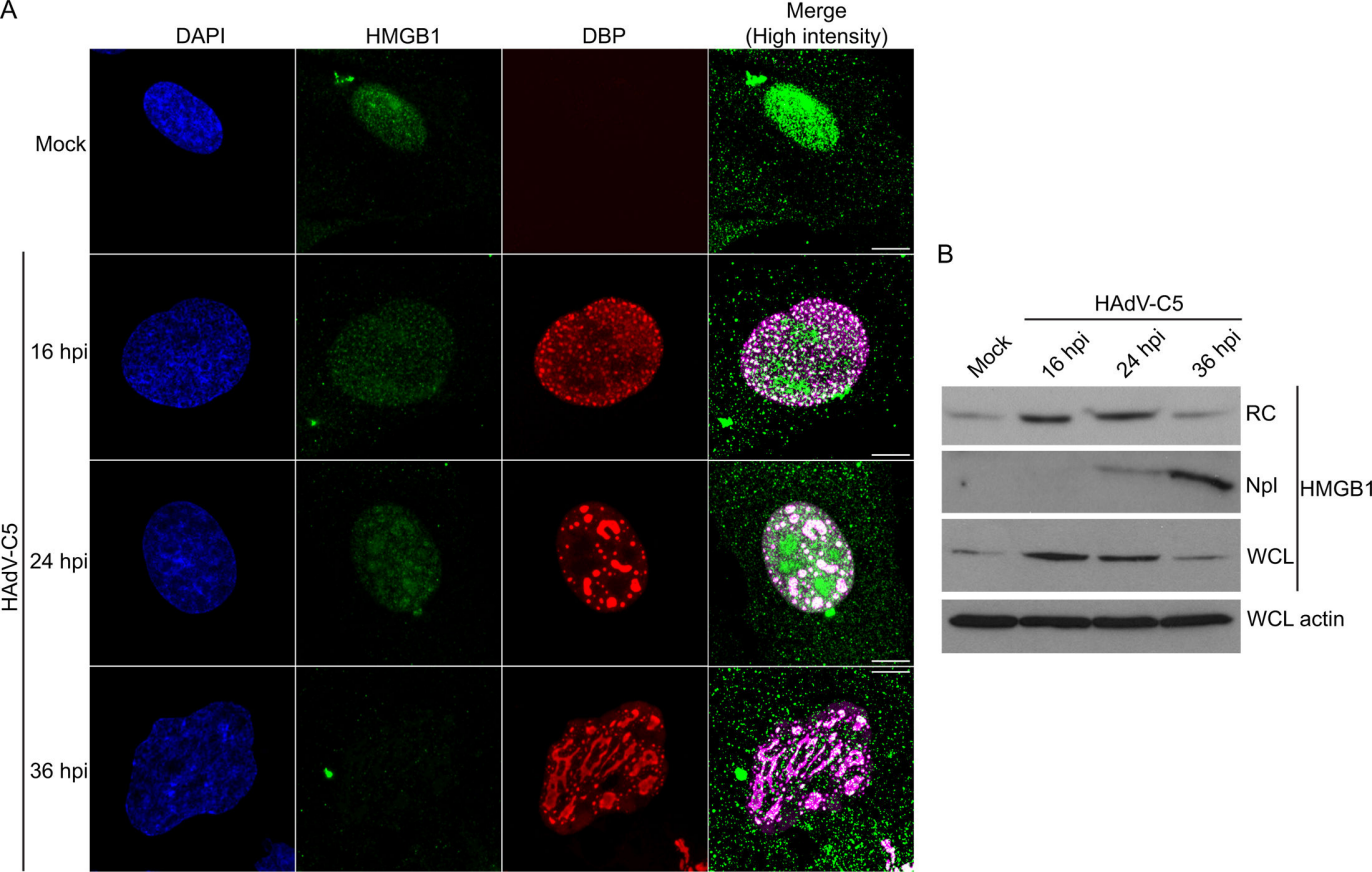
HMGB1 localizes to RCs. To validate its presence in RCs, fluorescence confocal microscopy was used to visualize the co-localization of HMGB1 with DBP-positive RCs at indicated time points post-infection (**A**) and corresponding immunoblot results of subnuclear and whole cell lysates (WCL) at 16, 24, and 36 hpi are shown (**B**). (**A**) HFFs infected with HAdV-C5 were fixed at 16, 24, or 36 hpi, stained for HMGB1 (green), DBP (red), and DNA (DAPI, blue), and visualized by confocal microscopy as z-stacks. The images are presented as maximum-intensity projections. The scale bars correspond to 10 µm. The *Merge* column shows the overlap of DBP (magenta) and HMGB1 (green), with the fluorescence intensity increased only with the purpose of enhancing the clarity of the colocalization areas. (**B**) The WCL and subcellular fractions of non-infected (mock) and HAdV-C5 infected HFFs were analyzed by immunoblot to detect HMGB1. Actin was used as the loading control.

Next, we turned our attention to proteins identified by our study as likely previously unknown RC members and that, based on their biological functions, may be important for RC formation or regulation of RC-associated activities or may act as restriction factors subverted in RCs. Notably, HMGB1, histone chaperones, and proteins that alter chromatin structure emerged as potential candidates in these capacities (Fig. 2B, cluster 1). Thus, we investigated the localization of HMGB1 in RCs during HAdV-C5 infection of HFFs. To validate its presence in RCs, fluorescence confocal microscopy was employed to visualize the co-localization of HMGB1 with DBP-positive RCs at the same time points post-infection used for the analysis of the RC proteome (16, 24, and 36 hpi; Fig. 6A). In mock-infected cells, HMGB1 was mainly diffusely distributed in the nucleoplasm as small foci and in one or two regions of the nucleus with the appearance of nucleoli (Fig. S3). During HAdV-C5 infection, HMGB-1 became re-localized to numerous nuclear regions with more densely grouped small foci. Most of these nuclear regions colocalized with RCs, whereas a few groups of foci seemed to exclude the DBP signal (Fig. 6A; Fig. S4 to S6). The HMGB1 protein appeared to decrease significantly in concentration by 36 hpi, suggesting that HAdV-C5 infection induces degradation of this protein. Immunoblot analyses of both subnuclear fractions and whole cell lysates at the same time points confirmed both an enrichment of HMGB1 in RCs and an overall decrease during the progression of HAdV-C5 infection (Fig. 6B).

**Fig 7:**
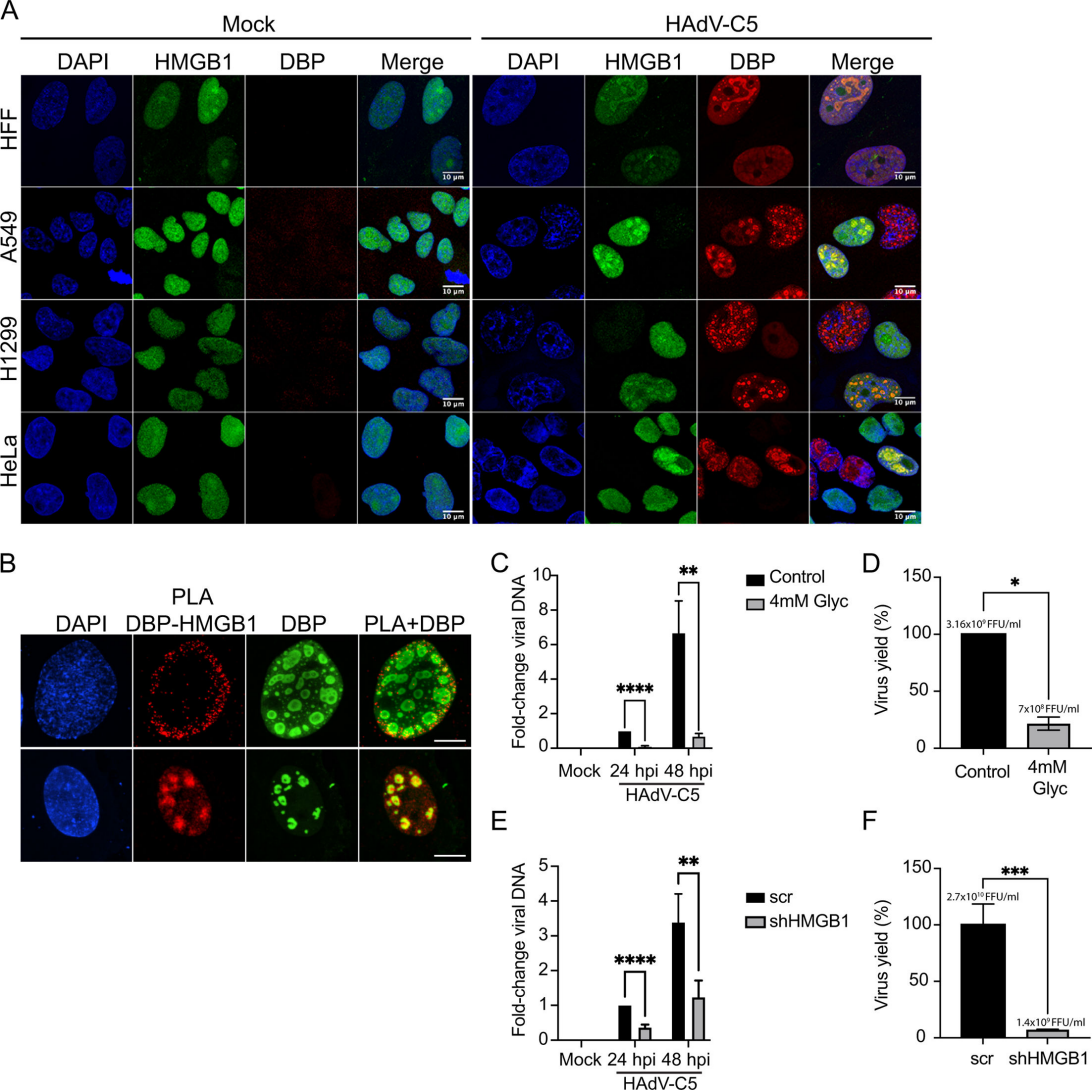
HMGB1 localizes to RCs in different cells, interacts with DBP, and is a proviral factor for HAdV-C5. (**A**) HFFs, A549, H1299, and HeLa cells mock-infected or infected with HAdV-C5 were fixed at 20 hpi. HMGB1 (green) was co-stained with DBP (red) and DNA (DAPI, blue). (**B**) PLA showing the interaction between DBP and HMGB1. A549 cells were infected with HAdV-C5 and fixed at 24 hpi. The cells were subjected to PLA using primary mAbs against HMGB1 and DBP, as described in the Materials and Methods section. Immunostaining for DBP was included as a reference for RCs. (**A and B**) All samples were visualized by confocal microscopy as z-stacks, and the images are presented as maximum-intensity projections. The scale bars correspond to 10 µm. PLA DBP-HMGB1: PLA signal for the interaction between DBP and HMGB1. (**C**) HAdV-C5 DNA levels were measured by quantitative PCR at 24 and 48 hpi in control cells or cells treated with 4 mM Glyc. (**D**) Virus yield was determined by quantitative DBP immunofluorescence of HAdV-C5-infected control A549 cells or HAdV-C5-infected cells treated with 4 mM Glyc at 48 hpi. (**E**) HAdV-C5 DNA levels were measured by quantitative PCR at 24 and 48 hpi, in cells stably expressing the scr or the HMGB1 shRNAs. (**F**) Virus yield was determined using quantitative DBP immunofluorescence. The samples were collected from either control (scr-) or shHMGB1-stably expressing A549 cells, which were infected with HAdV-C5 and harvested 48 hpi. (**C–F**) The results represent the averages of three independent experiments. Error bars indicate the standard error of the mean. *P < 0.05; **P < 0.01; ***P < 0.001; ****P < 0.0001. For viral DNA data, multiple *t* tests with a two-stage step-up method of Benjamini, Krieger and Yekutieli False Discovery Rate (FDR) approach were used. For virus yield data, unpaired *t* tests were used.

We then evaluated whether HMGB1 localization in RCs was specific to a particular cell type. To this end, we compared the localization of HMGB1 in HFFs with that in A549, H1299, and HeLa cell lines (Fig. 7A). The cells were mock-infected or infected with HAdV-C5 and fixed at 20 hpi. The colocalization of HMGB1 with DBP in RCs was confirmed by confocal microscopy (Fig. 7A). Similar to the findings in HFFs, we observed decreased levels of HMGB1 in all cell lines at late times post-infection (data not shown).

Previous research has demonstrated that the structural adenoviral protein VII can bind to HMGB1 in *in vitro* assays and sequesters HMGB1 on cellular chromatin. Consequently, the release of HMGB1 from cells is prevented, thereby blocking the immune response triggered by extracellular HMGB1 (60). As we observed that HMGB1 can localize to RCs, we hypothesized that DBP might also interact with HMGB1, causing its relocalization during HAdV-C5 infection. To test this, we performed proximity ligation assays (PLAs) to evaluate the *in situ* interactions between DBP and HMGB1 (Fig. 7B). With PLAs, positive interactions are detected within cells as fluorescent foci. We used mock-infected (Fig. S7) or HAdV-C5-infected A549 cells and fixed them at 24 hpi. These cells were then subjected to the PLA staining protocol, as described in Materials and Methods, and visualized using confocal microscopy. As shown in Fig. 7B, DBP indeed interacted with HMGB1, primarily in the nucleus. To correlate the interaction sites with RCs, we co-stained the cells with an anti-rabbit secondary antibody coupled to Alexa Fluor 488 to detect DBP. Surprisingly, DBP interacted with HMGB1 not only in RCs but also in other regions within the nucleoplasm (Fig. 7B). Similar results were observed for HFFs (data not shown). Controls for the PLA assays are shown in Fig. S7.

To determine the impact of HMGB1 on viral replication, we measured viral DNA levels and viral yield in the presence of the HMGB1 inhibitor glycyrrhizin (Glyc) and in A549 cells stably expressing a short-hairpin RNA (shRNA) against HMGB1 (shHMGB1; Fig. 7C through F). Glyc binds directly to HMGB1 with a dissociation constant of 150 µM and reduces its secretion but has only a mild effect on HMGB1 binding to nuclear DNA (61). We treated mock-infected or HAdV-C5-infected A549 cells with 200 µM (Fig. S8) or 4 mM Glyc (Fig. 7C and D). At 200 µM, only a mild reduction in viral DNA levels was detected at 48 hpi (Fig. S8A), but no significant difference was observed in the viral DNA levels at 24 hpi (Fig. S8A) or virus yield (Fig. S8B). However, when cells were treated with 4 mM Glyc, a concentration known to inhibit influenza virus polymerase activity (62), viral DNA levels were reduced by 13-fold at 24 hpi and 9.6-fold at 48 hpi (Fig. 7C). Additionally, virus production decreased by 78% (Fig. 7D). To confirm the effect of HMGB1 on HAdV-C5 infection, we transduced A549 cells with either scrambled (scr) or shHMGB1 and measured viral DNA and virus yield (Fig. 7E and F). The knockdown of HMGB1 did not affect A549 cell growth (Fig. S8C), and the knockdown efficiency was nearly 100% (Fig. S8D). Viral DNA levels decreased by 2.7-fold both at 24 hpi and 48 hpi (Fig. 7E), and virus yield decreased by 1 log (Fig. 7F). These results suggest that HMGB1 is recruited to viral RCs to promote viral replication.

**Fig 8:**
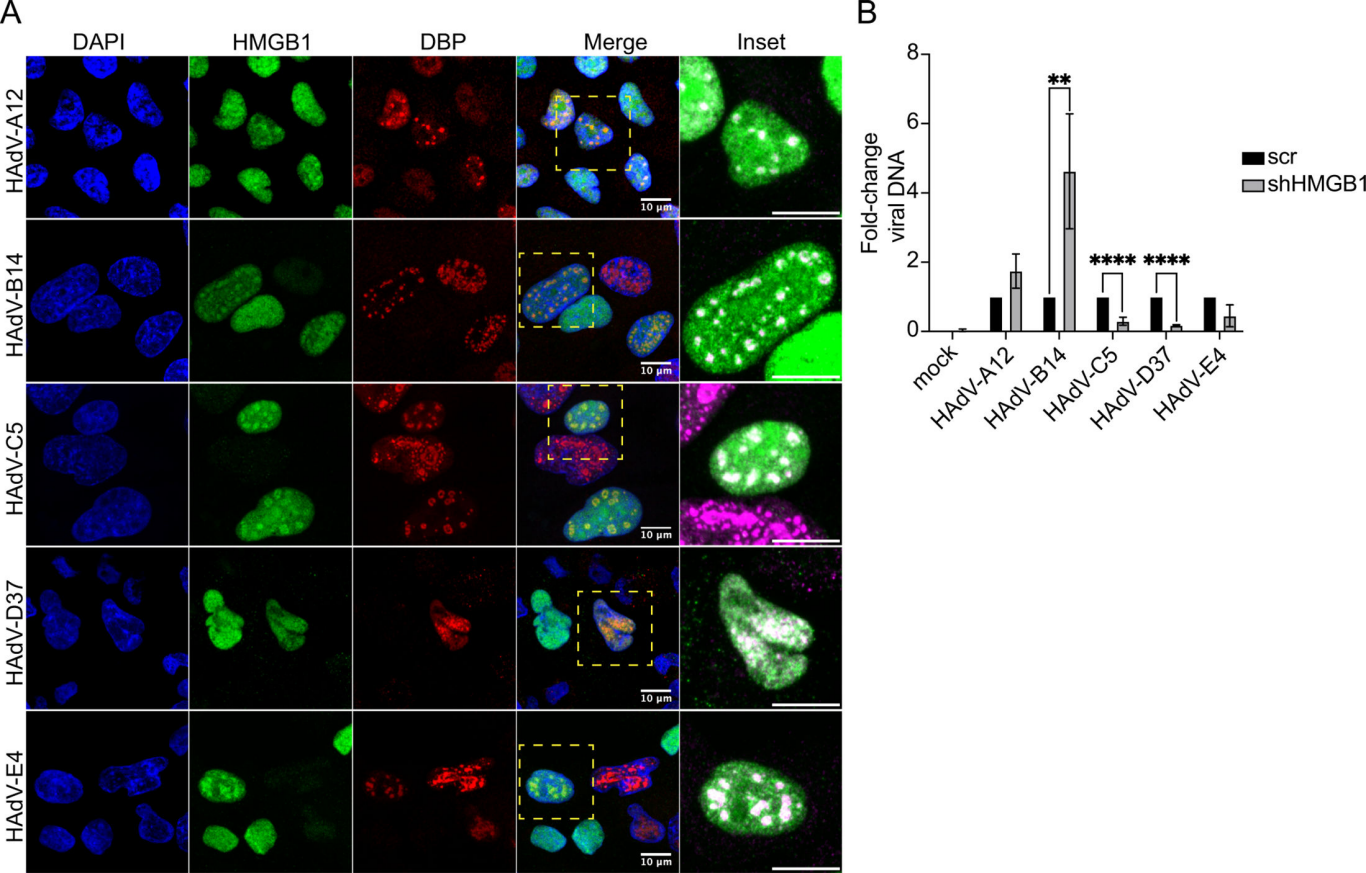
HMGB1 localizes to RCs across different HAdV species, with different impacts on viral DNA depending on the virus species. (**A**) A549 cells were infected with HAdV-A12, B14, C5, D37, or E4, and fixed at 20 hpi. HMGB1 was co-stained with DBP and DAPI. All samples were visualized by confocal microscopy as z-stacks, and the images are presented as maximum-intensity projections. The scale bars correspond to 10 µm. The *inset* column corresponds to the areas delimited by a dashed square in the *Merge* column. These insets show the overlap of DBP (magenta) and HMGB1 (green), with increased fluorescence intensity, only with the purpose of clarifying the regions of colocalization. (**B**) HAdV-A12, B14, -C5, -D37, and -E4 DNA levels were measured by quantitative PCR at 48 hpi, in cells stably expressing the scr or the HMGB1 shRNAs. Error bars indicate the standard error of the mean. **P < 0.01; ****P < 0.0001. Multiple *t* tests with a two-stage step-up method of Benjamini, Krieger, and Yekutieli FDR approach.

To determine whether the localization of HMGB1 in RCs is conserved among different HAdV species, we mock-infected or infected A549 cells with HAdV-A12, B14, C5, D37, or E4 (Fig. 8A). The cells were fixed at 20 hpi, stained for DBP and HMGB1, and counterstained with DAPI. The cells were then visualized by confocal microscopy. Our results consistently demonstrated the localization of HMGB1 in RCs in every case (Fig. 8A). Interestingly, in the absence of HMGB1, species C and D showed a significant decrease in viral DNA levels, whereas species B exhibited a nearly 5-fold increase (Fig. 8B). These findings suggest that although relocalization of HMGB1 to RCs is conserved among different HAdV species, its impact on viral replication may vary depending on the species.

**Fig 9:**
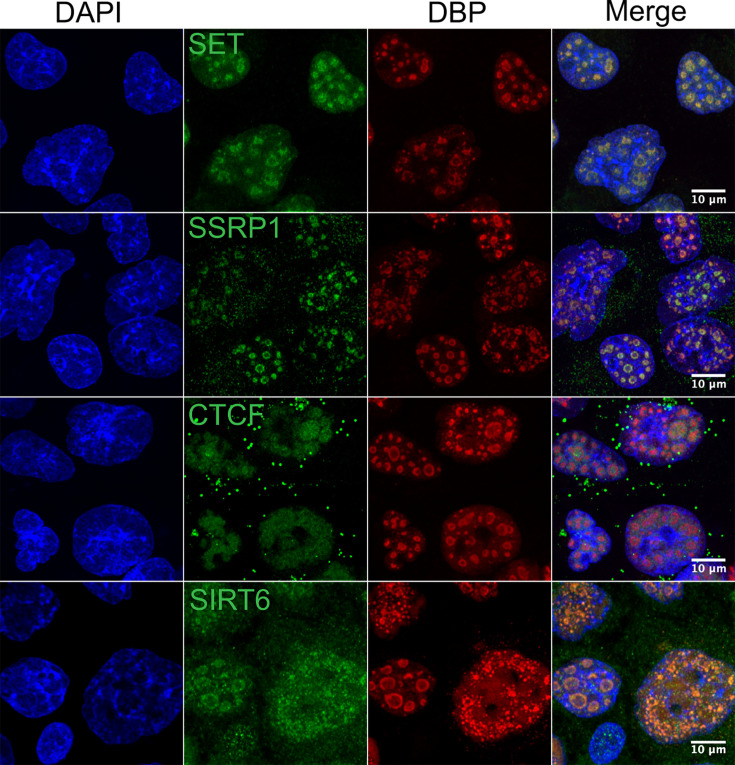
Proteins organizing chromatin architecture are relocalized to RCs during HAdV-C5 infection. HAdV-C5-infected A549 cells were fixed at 20 hpi. SET, SSRP1, CTCF, or SIRT6 (green) were co-stained together with DBP (red) and DNA (DAPI, blue) and visualized by confocal microscopy as z-stacks. The images are presented as maximum-intensity projections. The scale bars correspond to 10 µm.

Notably, several other histone chaperones or chromatin remodelers were also found to be associated with RCs in the TMT-MS analysis, some of which are known binding partners of HMGB1 or other high mobility group proteins, including SET, SSRP1, and CTCF (Fig. 1; Table S2) (63–65). Additionally, sirtuin 6 (SIRT6), a chromatin-binding cellular protein, was also identified (Table S1) (66). Confocal microscopy confirmed the localization of these cellular proteins to RCs in HAdV-C5-infected cells (Fig. 9). The distribution of the proteins in mock-infected cells is shown in Fig. S9.

## DISCUSSION

Formation of RCs in HAdV-infected cells is necessary for efficient genome replication and hence virus reproduction. Nevertheless, little is known about the functions of cellular proteins that are co-opted into these structures, or indeed, the mechanisms by which these infected cell-specific micro-environments form and change as the infectious cycle progresses. Here, we characterized the protein composition of HAdV RCs at different time points post-infection using TMT-MS of RC-enriched subnuclear fractions (39).

First, we aimed to explore the potential impact of proteins identified as associated with RCs at different times post-infection on distinct viral processes. Components of nucleoli and other subnuclear domains with similar dimensions to viral RCs were expected to be co-isolated in RC-subnuclear fractions (39). However, nucleoli and other nuclear domains are reorganized during HAdV-infection, and several of their components are relocalized to RCs (38, 67, 68). Therefore, it remained possible that specific and targeted recruitment of subnuclear domain components to RCs also takes place. These proteins were significantly enriched at 16 and 24 hpi (Fig. 2B, clusters 4 and 5; Fig. 3B, clusters 1 and 3). Hence, it will be interesting to study the interplay between RCs and nuclear domain components in detail.

Viral proteins and proteins participating in DNA synthesis and repair accumulated at 24 hpi and were significantly enriched at 36 hpi, correlating with increased viral DNA replication, transcription, and post-transcriptional RNA processing established around these times in RCs (reviewed in (24)). Interestingly, the minichromosome maintenance complex (MCM2-7) proteins, proteins that participate in DNA replication, were represented in RCs (Tables S2 and S3). MCM6 was recently shown to be associated with the viral genome (69). Further validation of MCM protein localization in RCs as well as determining their role in viral DNA should reveal valuable information about the regulation of viral DNA metabolism.

We observed that most proteins were equally distributed in RCs and Npl at 36 hpi (Fig. 2B). This finding was not unexpected: at this time point, RCs resemble a fibrillar mesh, occupying almost the entire cell-nucleus (Fig. 1A and reviewed in (24)). We have previously reported that early RCs exhibit liquid-like features, but with the progression of the viral replication cycle, the morphology of RCs increases in complexity, and these structures become both less dynamic and less fluid (26). These observations suggest that the procedure employed for the isolation of RC-enriched subnuclear fractions may be constrained by the morphological and biophysical characteristics of the RCs. Therefore, at the late time post-infection, it is difficult to differentiate and separate RC components from those of the rest of the nucleus.

In this study, we aimed to minimize false positives by producing a high-quality data set with a robust isolation procedure and stringent identification criteria. However, as with any proteomic-based discovery approach, a potential limitation is that the number of false discoveries cannot be accurately estimated without systematically testing a large number of the newly identified proteins. Several histone chaperones or proteins that alter chromatin structure were identified in RCs, among which HMGB1, SET, SSRP1, CTCF, and SIRT6 were validated by confocal microscopy (Fig. 6 to 9). Although a direct connection of SET to RCs has not been reported, it has been shown that the viral histone-like protein VII binds to SET. Such binding correlates with efficient viral early gene transcription, possibly as a result of inhibition of the DNA damage response activated upon nuclear import of the viral genome at an early stage of HAdV infection (70–72). CTCF plays a pivotal role as a nuclear component preserving the structural integrity of host chromatin architecture (73) and may interact with HMGB1 (65). CTCF was expected to be present in RCs because it promotes efficient viral DNA replication and late gene expression during HAdV-C5 replication (50). In this work, we established for the first time that CTCF is indeed localized to RCs (Fig. 9). Interestingly, Lang et al. demonstrated that CTCF is also recruited to the RCs of Herpes simplex 1 (HSV1) (34). SSRP1 is a high mobility group box (HMG)-containing protein and subunit of the FACT histone chaperone that can also modulate viral infections. In HIV infection, SSRP1 has been identified as a potential negative regulator of viral transcription (74). On the other hand, during HSV-1 infection, it has been observed that the viral protein ICP22 binds to and interacts with SSRP1, facilitating its recruitment to the viral genome and RCs. Such recruitment of SSRP1 by ICP22 has been correlated with enhanced viral transcription (75). Here, we show that SSRP1 is also recruited to HAdV-C5 RCs; however, whether SSRP1 has a positive or negative effect on adenoviral replication remains to be determined. SIRT6 is a multifunctional cellular protein that binds to chromatin and plays a crucial role in regulating double-stranded DNA repair and other chromatin functions (66). This protein belongs to a family of seven members, which are known antiviral factors (76). In future experiments, it would be interesting to investigate whether SIRT6 can restrict HAdV replication and if its localization in RCs serves as a mechanism to counteract this function.

HMGB1 is a multifunctional non-histone nuclear protein subjected to different PTMs (55). HMGB1 is mainly localized in the nucleus where it can act as a DNA chaperone to modulate DNA transcription, replication, recombination, and repair. Depending on the combination of PTMs, HMGB1 may be translocated into the cytoplasm to modulate autophagy or actively secreted or passively released into the extracellular space where it can act as a proinflammatory cytokine and activate the immune response (55).

Depending on the virus type, HMGB1 may associate with viral proteins or genomes to promote the infection, or in contrast, HMGB1 may stimulate the interferon response to restrict virus replication (77). During HAdV-C5-infection, HMGB1 may have both positive and negative roles. HMGB1, together with another member of the HMG family, HMGB2, stimulates the binding of the major late transcription factor (MLTF) to the adenoviral major late promoter (MLP), promoting its activation (78). The proinflammatory activity of HMGB1 is counteracted by the viral protein VII and the virus-associated RNAI (VA RNAI), which block its secretion. VA RNAI sequesters individual molecules of the interferon-inducible, double-stranded RNA-activated protein kinase (PKR) to prevent autoactivation of the enzyme and hence inflammasome activation. As the latter process leads to secretion of HMGB1, VA-RNAI can reduce extracellular concentrations of this protein indirectly (79, 80). Early during HAdV-C5 infection, protein VII sequesters HMGB1 in cellular chromatin, a change in location that correlates with a decrease in the secretion of HMGB1 (60). In the present work, we found that DBP also binds to HMGB1 (Fig. 7B) and that HMGB1 is a proviral factor for HAdV-C5 infection, promoting efficient accumulation in viral DNA and production of virus progeny (Fig. 7C through F). It is tempting to speculate that the interplay between the interaction of HMGB1 with DBP or with protein VII may dictate its recruitment to RCs.

In contrast to HAdV-C5 infection, the concentrations of HMGB1 in the cell culture supernatant showed an increase during the later stages of infection in HAdV-B7-infected A549 cells, where HMGB1 activated the immune response. However, HMGB1 also exerted a pro-viral effect on HAdV-B7 replication, as inhibition of this cellular protein resulted in a reduction of viral progeny (81). Intriguingly, we found that HMGB1 decreases the levels of HAdV-B14 DNA (Fig. 8B). The conservation of HMGB1 localization in RCs across different HAdV species (Fig. 8A) further underscores its importance for viral fitness. Whether HAdVs utilize or manipulate HMGB1 for particular viral processes, and whether this depends not only on the virus species but also on the virus type, requires further investigation.

Since cells and infection were not synchronized in these studies, it is expected to observe different phenotypes at the single-cell level (Fig. 7 and 8). However, the average concentrations of HMGB1 in HAdV-C5-infected HFFs (Fig. 6) and in A549-infected cells (data not shown) were increased at early times post-infection but greatly reduced at the latest time points examined. These observations suggest that intracellular HMGB1 might have different effects on RC-associated activities, depending on the stage of the viral replication cycle. We are now working on elucidating the role of HMGB1 on productive adenoviral infection, mainly at intermediate times when HMGB1 can be found in RCs and could regulate RC-associated activities.

Our work sets a precedent for future studies on viral RCs that should improve the understanding of how these viral-induced microenvironments are assembled and what components and activities are modulated within them. Moreover, since compartmentalization of the infected cell is a general strategy established by different viruses, targeting key RC components could be used to develop antiviral therapies.

## MATERIALS AND METHODS

### Cells and viruses

Primary human foreskin fibroblasts (HFFs; kindly provided by Jesús Santa-Olalla (School of Medicine—UAEM)), and the cell lines A549, H1299 and HeLa cells were maintained in monolayer cultures in Dulbecco’s modified Eagle’s medium (Gibco) supplemented with 10% fetal bovine serum (Gibco), 100 U/mL penicillin, and 100 µg/mL streptomycin (Gibco). We used H5*pg*4100 as the HAdV-C5 wild-type (wt) virus (82), along with HAdV prototype strains HAdV-A12, HAdV-B14, HAdV-D37, and HAdV-E4 (83). For all experiments, HFFs were infected with an MOI of 30 foci-forming units (FFU) per cell (FFU/cell), and all the used cell lines were infected using an MOI of 10 FFU/cell. As indicated, cells were grown in the presence of 200 µM or 4 mM Glyc (Sigma-Aldrich). The number of viable cells was obtained using a Neubauer chamber and trypan blue dye, as described before (52).

### Cell fractionation

In total, 1 × 10^7^ mock-infected or HAdV-C5-infected cells were harvested at 16, 24, and 36 hpi and fractionated following a previously published protocol (38, 39). Briefly, cells were lysed, and subnuclear fractions were isolated via sucrose cushions and centrifugation. The nucleoplasmic fractions (Npl) and the pellets containing the RC-enriched fraction or the nucleolar fraction (Nlo; in mock-infected cells) were stored at −80°C until further use.

### Dynamic light scattering (DLS) of RCs

RC-enriched fractions were isolated from mock-infected or HAdV-C5 infected HFFs at 24 and 36 hpi. The hydrodynamic diameter of the particles contained within each fraction was measured using the Malvern Zetasizer Nano SP at 4°C. Two measurements were performed for each sample, with 12 reads of 10 s per each measurement. The S2 Buffer (buffer used to resuspend the RC fractions (38, 39)) viscosity coefficient at 4°C was calculated using a rheometer and was estimated at 1.88 Ns/m^2^. The refractive index calculated for S2 was 1.3504. The hydrodynamic diameter was calculated by the Stokes-Einstein equation, and the values of intensity per size were plotted. The results represent the average of three biological replicates.

### TMT-MS analysis

The proteomic analysis of Npl and RC-enriched fractions from the same number of mock-infected or HAdV-C5-infected HFFs was performed in three biological replicates. Approximately 400 µg of protein for each sample was resuspended in lysis buffer (0.1 M ammonium bicarbonate, 2.5% [wt/vol] sodium deoxycholate, 10 mM pH neutral tris(2-carboxyethyl) phosphine [TCEP], 40 mM chloroacetamide [CAM]). The proteins were subsequently precipitated with methanol:chloroform solution and digested with trypsin. Protein concentration was measured by the BCA protein assay, and 100 µg of digested peptide was processed for TMT-MS quantification as described previously (41). A master mix (MM, Table S1), a mixture of all samples, was included both for the nucleoplasm and RC samples, (NP MM and RC MM, respectively) to normalize across experiments.

### Protein identification, quantification, and statistical analyses

TMT-MS data were extracted using MaxQuant version 1.5.2.8 (84) and the human subset of the UniProt protein sequence database, appended with HAdV-C5 sequences and common sample contaminants for protein identification and quantification. The search parameters were as follows: trypsin as the enzyme with a maximum of two missed cleavages permitted. The mass tolerance was set to 10 ppm for precursor ions. Carbamidomethyl was set as the fixed modification. The intensities of all TMT reporter ions from peptide spectrum matches were extracted. Peptide groups were considered for quantification based on their uniqueness (unique peptides) and in accordance with the principle of parsimony. The final criterion was at least two peptide matches. Three biological replicates were carried out to evaluate the reproducibility of the identification. The PCC was used to assess the correlation between the replicates and groups. Protein abundances were therein calculated as the average among triplicates of the summed TMT intensities. For the calculation of protein fold-change abundances, data were log2-transformed following median normalization to satisfy the requirement of a normal distribution. Unpaired Student’s *t*-tests, assuming unequal variance, were employed to assess significance levels, with *P*-values adjusted using the Benjamini and Hochberg (BH) method for multiple comparisons. A false discovery rate (FDR) of 1% was selected as the criterion for significance.

### Bioinformatics analyses

Exploratory analysis using a principal component analysis (PCA) and box plots was done using R environment (v 4.2.3) (85). The protein abundance was used to determine the significant enrichment of proteins from RC fractions using the following three different approaches: (i) proteins present in all the three biological replicates from the RC-enriched subnuclear fractions; (ii) a multivariate analysis comparing the proteins from HAdV-C5-infected cells with that of mock-infected cells (mock) at the three times post-infection; and (iii) a univariate analysis to compare the proteins of RC fractions against Npl fractions for each time post-infection independently. These analyses were done with the Limma package using a threshold of >2 peptides per protein without missing values by group. Unpaired Student’s *t*-tests were conducted, and *P*-values were adjusted using the BH method for multiple comparisons. A standard cutoff *P*-value of ≤0.05, with a false discovery rate (FDR) of 1%, was employed as the threshold for significance. The resulting enriched proteins from each analysis were used to generate heatmaps. The heatmaps were based on Euclidean distance and were divided into clusters based on the optimal number of clusters determined by the *gap* statistic, using default parameters in the clusGap-function from the cluster package (v2.1.6, (86)). Venn diagrams were created with ggVennDiagram (v 1.2.2) and ggplot (v3.4.1). Proteins without significant values at any time point were denoted as zero for visualization purposes. Gene ontology (GO) enrichment analysis and pathway enrichment analysis were carried out using g:Profiler (v 0.2.2) and KEGG pathways, respectively.

To validate previously reported data on proteins of interest, a targeted search was conducted, and the results were presented as a heatmap. To identify protein-protein interactions, both functional and physical, between the significant selected candidate proteins, we used the STRING database (https://string-db.org/(46)). The interaction networks were visualized using Cytoscape (version: 3.9.1).

### Immunofluorescence microscopy

Cells were seeded on coverslips and mock-infected or HAdV-C5 wt-infected and fixed at the specified time points. Next, the cells were processed for IF microscopy as described previously (Table 1) (22). The coverslips were mounted on glass slides in Fluoroshield with 1,4-diazabicyclo [2.2.2] octane (Sigma-Aldrich). Cells were imaged using an inverted confocal scanning laser microscope (Nikon A1R HD25 equipped with a Nikon 60× oil immersion NA 1.40 objective). Images were acquired as Z-stacks spaced every 0.3 µm and processed using Fiji (87). Image acquisition and processing were adjusted identically for compared image sets. Figures are presented as maximum z-projections. For the *Merge (high intensity*) columns in Fig. 6 and Fig. S4 to S6 and the *Inset* column in Fig. 8, the fluorescence intensity was increased only with the purpose of enhancin the clarity of the colocalization areas.

**TABLE 1:** Antibodies^*a*^

	Antibody	Purpose	Dilution	Reference or source
Primary antibodies	Anti-DBP mouse mAb B6-8	IF	1:5,000	(88)
	Anti-DBP rabbit pAb	IF, PLA	1:5,000	Ann E. Tollefson, St. Louis University (USA)
	Anti-HMGB1 mouse mAb W-18	IF, WB, PLA	1:200	Santa Cruz Biotechnology
	Anti-actin mouse mAb A-5441	WB	1:10,000	Sigma-Aldrich
	Anti-RPA2 mouse mAb 9H8	IF	1:200	Santa Cruz Biotechnology
	Anti-TopBP1 mouse mAb B-7	IF	1:200	Santa Cruz Biotechnology
	Anti-IVa2 mouse mAb 9F4 and 6C9	IF	1:200	Carmen San Martín, Centro Nacional de Biotecnología (Spain)
	Anti-USP7 rabbit pAb	IF, PLA	1:500	Abcam
	Anti-SET mouse mAb F-9	IF	1:200	Santa Cruz Biotechnology
	Anti-SSRP1 mouse mAb D-7	IF	1:200	Santa Cruz Biotechnology
	Anti-CTCF mouse mAb	IF	1:200	Santa Cruz Biotechnology
	Anti-SIRT6 mouse mAb 2G1H1	IF	1:200	Santa Cruz Biotechnology
	Anti-53BP1 mouse mAb E-10	PLA	1:200	Santa Cruz Biotechnology
Secondary antibodies	Anti-mouse horseradish peroxidase (HRP)-conjugated antibody	WB	1:10,000	Jackson ImmunoResearch
	Anti-mouse AlexaFluor 488	IF	1:1,500	Thermo Scientific
	Anti-rabbit AlexaFluor 488	IF	1:1,500	Thermo Scientific
	Anti-mouse AlexaFluor 555	IF	1:1,500	Thermo Scientific

^*a*^ mAb, monoclonal antibody; pAb, polyclonal antibody; WB, western (immuno-) blotting; IF, immunofluorescence microscopy; PLA, proximity ligation assay.

### Immunoblotting

To analyze HMGB1 and actin steady-state concentrations, subnuclear fractions and whole cell lysates (WCL) from mock-infected and HAdV-C5-infected cells at 16, 24, and 36 hpi were obtained. Protein concentration was measured spectrophotometrically using the Bradford reagent (Bio-Rad). For immunoblotting, 25 (actin) or 50 (HMGB1) µg of total protein lysates were analyzed by SDS-PAGE, transferred onto nitrocellulose membranes (GE Healthcare), and blocked overnight with 5% non-fat milk. The next day, the membranes were washed and incubated for at least 2 h at 4°C with the indicated primary antibodies (Table 1). After successive washes with PBS/0.1% Tween20 (PBS-T), the membranes were incubated with secondary antibodies coupled to HRP (Table 1) for at least 45 min at 4°C and thoroughly washed with PBS-T. Membranes were developed by enhanced chemiluminescence as recommended by the manufacturer (Pierce, Thermo Scientific) on X-ray films (Fujifilm).

### DNA isolation and quantitative PCR

To determine viral DNA levels, A549 cells were mock-infected or infected with HAdV-C5, -A12, -B14, -D37, or E4. HAdV-C5-infected cells were harvested at 24 and 48 hpi, whereas cells infected with other HAdV species were harvested at 48 hpi. The QIAamp DNA Mini Kit (Qiagen) was used to isolate total DNA following the manufacturer’s protocol. Quantitative PCRs (qPCRs) were performed on the DNA samples using a Rotor-Gene 6000 (Corbett Life Sciences), SYBR Green chemistry, and the following E2A primers: primers specific for HAdV-C5 (forward primer 5′-GGT CTG GGC GTT AGG ATA CA-3′; reverse primer 5′-CAA TCA GTT TTC CGG CAA GT-3′); HAdV-A12 (forward primer 5′-TTT ATG CTG GCC GAC TAC CC-3′; reverse primer 5′-TTT ACA CAG TTG ACG CCC CA-3′); HAdV-B14 (forward primer 5′-GAG AAT CCC GAG CGA ACC AA-3′; reverse primer 5′-CAT CAT TCA CAC AGC AGC GG-3′); HAdV-D37 (forward primer 5′-ACC AAG CGC CGA AAG TAT CA-3′; reverse primer 5′-TCT TCG TCG GGG TCT ACC TT-3′); HAdV-E4 (forward primer 5′-AAC TTC CAA CTG ATG CCC GA-3′; reverse primer 5′-TCA TGG TCG TCA GGG TCT TG-3′); GAPDH (forward primer 5′-ACC ACA GTC CAT GCC ATC AC-3′; and reverse primer 5′-TCC ACC ACC CTG TTG CTG TA-3′).

### Generation of cells expressing the shHMGB1

To establish stable HMGB1 scr or HMGB1 knockdown HFFs or A549 cells, these cells were seeded onto 6-well dishes and transduced with lentiviral particles produced with the plasmid encoding an shRNA against HMGB1 (target sequence CCC AGA TGC TTC AGT CAA CTT; TRCN0000018934, MISSION shRNA products, Sigma-Aldrich) or the non-target shRNA as control (scr; target sequence: CCG GCA ACA AGA TGA AGA GCA CCA ACT CGA GTT GGT GCT CTT CAT CTT GTT GTT TTT; SHC002, MISSION shRNA products, Sigma-Aldrich) and a puromycin resistance gene for the selection of cells stably expressing shRNA. Two days after transduction, fresh media containing 2–3 µg/mL of puromycin (Sigma-Aldrich) was added to select the stably transduced cells. The cells were then propagated with fresh puromycin-containing media added each time. The cells were infected and processed as described above.

### Virus yield quantification

To determine virus yield, HFFs or A549 cells (shHMGB1-transduced or Glyc-treated) were infected with HAdV-C5 and harvested at 48 hpi. Cells were lysed by three freeze-thaw cycles. The cell lysate supernatants were serially diluted in DMEM for infection of A549 cells, and the virus yield was determined by immunofluorescence microscopy and counting cells expressing DBP at 24 hpi, as described previously (89, 90).

### Proximity ligation assays

A549 cells were grown on Ibidi chambered coverslips (µ-Slide 8 Well High Glass Bottom, 80807), mock-infected or infected with HAdV-C5. Cells were fixed with paraformaldehyde (PFA; 4% (vol/vol) in PBS) at room temperature (RT) for 10 min. PFA-fixed cells were permeabilized with phosphate-buffered saline (PBS) supplemented with 0.5% Triton X-100 for 5 min at RT and blocked with Tris-buffered saline-BG (TBS-BG; BG represents 5% (wt/vol) BSA and 5% (wt/vol) glycine) for 1 h at RT. The cells were then incubated with a mixture of mouse anti-HMGB1 and rabbit anti-DBP antibodies (Table 1) and further subjected to PLA with the Duolink *In Situ* Detection Reagents Red kit (Sigma-Aldrich) according to the manufacturer’s instructions. Where indicated, the secondary antibody anti-rabbit AlexaFluor 488 was present in the mixture of PLA probes to counterstain DBP. Cells were kept in PBS and imaged by confocal scanning laser microscopy. As negative controls for the PLAs, PLA with 53BP1 as a protein not binding to DBP as well as PLA of DBP with HMGB1 in mock-infected cells were used. As a control for fluorescence bleed-through between the PLA signal of DBP with HMGB1 and visualization of DBP, no counterstaining of DBP (no secondary antibody anti-rabbit AlexaFluor 488) was included. As a positive control for the assay, a PLA was performed for DBP and its already known binding protein USP7 (36) (Fig. S7).

### Statistical analyses for PCR and virus yield assays

All statistical analyses were performed with GraphPad Prism v9 (GraphPad Software). Specific information on the statistical tests is provided in the respective figure legends. Data were considered significantly different if the *P* value was ≤ 0.05.

## Data Availability

The TMT-MS proteomics data have been deposited to the ProteomeXchange Consortium via the PRIDE partner repository (91) with the dataset identifier PXD051745. Additional data resulting from the analyses are presented as supplementary tables.

## SUPPLEMENTAL MATERIAL

Supplemental Figures mbio.02144-24-s0001.pdf Fig. S1 to S9.

Table S1 mbio.02144-24-s0002.xlsx Total proteins identified in RC and Npl fractions from mock-infected and HAdV-C5-infected cells at different time points post-infection.

Table S2 mbio.02144-24-s0003.xlsx Cluster analysis of the RC proteome at different time points post-infection.

Table S3 mbio.02144-24-s0004.xlsx Cluster analysis of proteins in RCs or Npl from HAdV-C5 infected cells relative to mock-infected cells.

Table S4 mbio.02144-24-s0005.xlsx Cluster analysis of proteins in RCs relative to Npl.

ASM does not own the copyrights to Supplemental Material that may be linked to, or accessed through, an article. The authors have granted ASM a non-exclusive, world-wide license to publish the Supplemental Material files. Please contact the corresponding author directly for reuse.

## References

[1] Casadevall A, Fang FC. 2020. The intracellular pathogen concept. Mol Microbiol 113:541–545. doi:10.1111/mmi.14421 31762116

[2] Knipe DM, Prichard A, Sharma S, Pogliano J. 2022. Replication compartments of eukaryotic and bacterial DNA viruses: common themes between different domains of host cells. Annu Rev Virol 9:307–327. doi:10.1146/annurev-virology-012822-125828 36173697 PMC10311714

[3] Netherton C, Moffat K, Brooks E, Wileman T. 2007. A guide to viral inclusions, membrane rearrangements, factories, and viroplasm produced during virus replication. Adv Virus Res 70:101–182. doi:10.1016/S0065-3527(07)70004-0 17765705 PMC7112299

[4] Netherton CL, Wileman T. 2011. Virus factories, double membrane vesicles and viroplasm generated in animal cells. Curr Opin Virol 1:381–387. doi:10.1016/j.coviro.2011.09.008 22440839 PMC7102809

[5] Nevers Q, Albertini AA, Lagaudrière-Gesbert C, Gaudin Y. 2020. Negri bodies and other virus membrane-less replication compartments. Biochim Biophys Acta Mol Cell Res 1867:118831. doi:10.1016/j.bbamcr.2020.118831 32835749 PMC7442162

[6] Schmid M, Speiseder T, Dobner T, Gonzalez RA. 2014. DNA virus replication compartments. J Virol 88:1404–1420. doi:10.1128/JVI.02046-13 24257611 PMC3911613

[7] Wolff G, Melia CE, Snijder EJ, Bárcena M. 2020. Double-membrane vesicles as platforms for viral replication. Trends Microbiol 28:1022–1033. doi:10.1016/j.tim.2020.05.009 32536523 PMC7289118

[8] Ivanovic T, Boulant S, Ehrlich M, Demidenko AA, Arnold MM, Kirchhausen T, Nibert ML. 2011. Recruitment of cellular clathrin to viral factories and disruption of clathrin-dependent trafficking. Traffic 12:1179–1195. doi:10.1111/j.1600-0854.2011.01233.x 21736684 PMC3575638

[9] Neufeldt CJ, Joyce MA, Van Buuren N, Levin A, Kirkegaard K, Gale M Jr, Tyrrell DLJ, Wozniak RW. 2016. The hepatitis C virus-induced membranous web and associated nuclear transport machinery limit access of pattern recognition receptors to viral replication sites. PLoS Pathog 12:e1005428. doi:10.1371/journal.ppat.1005428 26863439 PMC4749181

[10] Paul D, Bartenschlager R. 2015. Flaviviridae replication organelles: oh, what a tangled web we weave. Annu Rev Virol 2:289–310. doi:10.1146/annurev-virology-100114-055007 26958917

[11] Borodavka A, Acker J. 2023. Seeing biomolecular condensates through the lens of viruses. Annu Rev Virol 10:163–182. doi:10.1146/annurev-virology-111821-103226 37040799

[12] Jean Beltran PM, Cook KC, Cristea IM. 2017. Exploring and exploiting proteome organization during viral infection. J Virol 91:e00268-17. doi:10.1128/JVI.00268-17 28679763 PMC5571249

[13] Cook KC, Tsopurashvili E, Needham JM, Thompson SR, Cristea IM. 2022. Restructured membrane contacts rewire organelles for human cytomegalovirus infection. Nat Commun 13:4720. doi:10.1038/s41467-022-32488-6 35953480 PMC9366835

[14] DiMaio D. 2019. Small size, big impact: how studies of small DNA tumour viruses revolutionized biology. Philos Trans R Soc Lond B Biol Sci 374:20180300. doi:10.1098/rstb.2018.0300 30955494 PMC6501907

[15] Tessier TM, Dodge MJ, MacNeil KM, Evans AM, Prusinkiewicz MA, Mymryk JS. 2021. Almost famous: human adenoviruses (and what they have taught us about cancer). Tumour Virus Res 12:200225. doi:10.1016/j.tvr.2021.200225 34500123 PMC8449131

[16] Gao J, Mese K, Bunz O, Ehrhardt A. 2019. State-of-the-art human adenovirus vectorology for therapeutic approaches. FEBS Lett 593:3609–3622. doi:10.1002/1873-3468.13691 31758807

[17] Barry MA, Rubin JD, Lu SC. 2020. Retargeting adenoviruses for therapeutic applications and vaccines. FEBS Lett 594:1918–1946. doi:10.1002/1873-3468.13731 31944286 PMC7311308

[18] Coughlan L, Kremer EJ, Shayakhmetov DM. 2022. Adenovirus-based vaccines-a platform for pandemic preparedness against emerging viral pathogens. Mol Ther 30:1822–1849. doi:10.1016/j.ymthe.2022.01.034 35092844 PMC8801892

[19] Lion T. 2019. Adenovirus persistence, reactivation, and clinical management. FEBS Lett 593:3571–3582. doi:10.1002/1873-3468.13576 31411731

[20] Komatsu T, Quentin-Froignant C, Carlon-Andres I, Lagadec F, Rayne F, Ragues J, Kehlenbach RH, Zhang W, Ehrhardt A, Bystricky K, Morin R, Lagarde JM, Gallardo F, Wodrich H. 2018. In vivo labelling of adenovirus DNA identifies chromatin anchoring and biphasic genome replication. J Virol 92:e00795-18. doi:10.1128/JVI.00795-18 29997215 PMC6146703

[21] Halbert DN, Cutt JR, Shenk T. 1985. Adenovirus early region 4 encodes functions required for efficient DNA replication, late gene expression, and host cell shutoff. J Virol 56:250–257. doi:10.1128/jvi.56.1.250-257.1985 4032537 PMC252513

[22] Gonzalez R, Huang W, Finnen R, Bragg C, Flint SJ. 2006. Adenovirus E1B 55-kilodalton protein is required for both regulation of mRNA export and efficient entry into the late phase of infection in normal human fibroblasts. J Virol 80:964–974. doi:10.1128/JVI.80.2.964-974.2006 16378998 PMC1346875

[23] Sohn SY, Hearing P. 2019. Adenoviral strategies to overcome innate cellular responses to infection. FEBS Lett 593:3484–3495. doi:10.1002/1873-3468.13680 31721176 PMC6928427

[24] Hidalgo P, Gonzalez RA. 2019. Formation of adenovirus DNA replication compartments. FEBS Lett 593:3518–3530. doi:10.1002/1873-3468.13672 31710378

[25] Bertzbach LD, Seddar L, von Stromberg K, Ip W-H, Dobner T, Hidalgo P. 2024. The adenovirus DNA-binding protein DBP. J Virol 98:e0188523. doi:10.1128/jvi.01885-23 38197632 PMC10878046

[26] Hidalgo P, Pimentel A, Mojica-Santamaría D, von Stromberg K, Hofmann-Sieber H, Lona-Arrona C, Dobner T, González RA. 2021. Evidence that the adenovirus single-stranded DNA binding protein mediates the assembly of biomolecular condensates to form viral replication compartments. Viruses 13:1778. doi:10.3390/v13091778 34578359 PMC8473285

[27] Voelkerding K, Klessig DF. 1986. Identification of two nuclear subclasses of the adenovirus type 5-encoded DNA-binding protein. J Virol 60:353–362. doi:10.1128/JVI.60.2.353-362.1986 2945931 PMC288900

[28] Charman M, Herrmann C, Weitzman MD. 2019. Viral and cellular interactions during adenovirus DNA replication. FEBS Lett 593:3531–3550. doi:10.1002/1873-3468.13695 31764999 PMC6928415

[29] Charman M, Weitzman MD. 2020. Replication compartments of DNA viruses in the nucleus: location, location, location. Viruses 12:151. doi:10.3390/v12020151 32013091 PMC7077188

[30] Fricke J, Koo LY, Brown CR, Collins PL. 2013. P38 and OGT sequestration into viral inclusion bodies in cells infected with human respiratory syncytial virus suppresses MK2 activities and stress granule assembly. J Virol 87:1333–1347. doi:10.1128/JVI.02263-12 23152511 PMC3554139

[31] Helbig KJ, Eyre NS, Yip E, Narayana S, Li K, Fiches G, McCartney EM, Jangra RK, Lemon SM, Beard MR. 2011. The antiviral protein viperin inhibits hepatitis C virus replication via interaction with nonstructural protein 5A. Hepatology 54:1506–1517. doi:10.1002/hep.24542 22045669 PMC3207276

[32] Jobe F, Simpson J, Hawes P, Guzman E, Bailey D. 2020. Respiratory syncytial virus sequesters NF-κB subunit p65 to cytoplasmic inclusion bodies to inhibit innate immune signaling. J Virol 94:e01380-20. doi:10.1128/JVI.01380-20 32878896 PMC7592213

[33] Erickson KD, Garcea RL. 2019. Viral replication centers and the DNA damage response in JC virus-infected cells. Virology (Auckl) 528:198–206. doi:10.1016/j.virol.2018.12.014 30811999

[34] Lang FC, Li X, Vladmirova O, Li ZR, Chen GJ, Xiao Y, Li LH, Lu DF, Han HB, Zhou JM. 2015. Selective recruitment of host factors by HSV-1 replication centers. Dongwuxue Yanjiu 36:142–151. 26018857 PMC4790689

[35] Rice SA, Klessig DF. 1985. Isolation and analysis of adenovirus type 5 mutants containing deletions in the gene encoding the DNA-binding protein. J Virol 56:767–778. doi:10.1128/JVI.56.3.767-778.1985 3864995 PMC252647

[36] Boddin J, Ip W-H, Wilkens B, von Stromberg K, Ching W, Koyuncu E, Bertzbach LD, Dobner T. 2022. A single amino acid switch in the adenoviral DNA binding protein abrogates replication center formation and productive viral infection. MBio 13:e0014422. doi:10.1128/mbio.00144-22 35254132 PMC9040859

[37] Chahal JS, Qi J, Flint SJ. 2012. The human adenovirus type 5 E1B 55 kDa protein obstructs inhibition of viral replication by type I interferon in normal human cells. PLoS Pathog 8:e1002853. doi:10.1371/journal.ppat.1002853 22912576 PMC3415460

[38] Hidalgo P, Anzures L, Hernández-Mendoza A, Guerrero A, Wood CD, Valdés M, Dobner T, Gonzalez RA. 2016. Morphological, biochemical, and functional study of viral replication compartments isolated from adenovirus-infected cells. J Virol 90:3411–3427. doi:10.1128/JVI.00033-16 26764008 PMC4794690

[39] Hidalgo P, Gonzalez RA. 2015. Isolation of viral replication compartment-enriched sub-nuclear fractions from adenovirus-infected normal human cells. J Vis Exp 105:53296. doi:10.3791/53296 PMC4692707 26649626

[40] Hidalgo P, Garcés Y, Mundo E, López RE, Bertzbach LD, Dobner T, González RA. 2022. E1B-55K is a phosphorylation-dependent transcriptional and post-transcriptional regulator of viral gene expression in HAdV-C5 infection. J Virol 96:e0206221. doi:10.1128/jvi.02062-21 35019711 PMC8906433

[41] Jean Beltran PM, Mathias RA, Cristea IM. 2016. A portrait of the human organelle proteome in space and time during cytomegalovirus infection. Cell Syst 3:361–373. doi:10.1016/j.cels.2016.08.012 27641956 PMC5083158

[42] Thompson A, Schäfer J, Kuhn K, Kienle S, Schwarz J, Schmidt G, Neumann T, Johnstone R, Mohammed AKA, Hamon C. 2003. Tandem mass tags: a novel quantification strategy for comparative analysis of complex protein mixtures by MS/MS. Anal Chem 75:1895–1904. doi:10.1021/ac0262560 12713048

[43] Komatsu T, Robinson DR, Hisaoka M, Ueshima S, Okuwaki M, Nagata K, Wodrich H. 2016. Tracking adenovirus genomes identifies morphologically distinct late DNA replication compartments. Traffic 17:1168–1180. doi:10.1111/tra.12429 27492875

[44] Garcés Y, Guerrero A, Hidalgo P, López RE, Wood CD, Gonzalez RA, Rendón-Mancha JM. 2016. Automatic detection and measurement of viral replication compartments by ellipse adjustment. Sci Rep 6:36505. doi:10.1038/srep36505 27819325 PMC5098162

[45] Murti KG, Davis DS, Kitchingman GR. 1990. Localization of adenovirus-encoded DNA replication proteins in the nucleus by immunogold electron microscopy. J Gen Virol 71 (Pt 12):2847–2857. doi:10.1099/0022-1317-71-12-2847 2273386

[46] von Mering C, Jensen LJ, Kuhn M, Chaffron S, Doerks T, Kruger B, Snel B, Bork P. 2007. STRING 7--recent developments in the integration and prediction of protein interactions. Nucleic Acids Res 35:D358–D362. doi:10.1093/nar/gkl825 17098935 PMC1669762

[47] Cardoso FM, Kato SEM, Huang W, Flint SJ, Gonzalez RA. 2008. An early function of the adenoviral E1B 55 kDa protein is required for the nuclear relocalization of the cellular p53 protein in adenovirus-infected normal human cells. Virology (Auckl) 378:339–346. doi:10.1016/j.virol.2008.06.016 18632130

[48] Yang H, Zheng Z, Zhao LY, Li Q, Liao D. 2012. Downregulation of Mdm2 and Mdm4 enhances viral gene expression during adenovirus infection. Cell Cycle 11:582–593. doi:10.4161/cc.11.3.19052 22262167 PMC3315096

[49] Komatsu T, Nagata K. 2012. Replication-uncoupled histone deposition during adenovirus DNA replication. J Virol 86:6701–6711. doi:10.1128/JVI.00380-12 22496236 PMC3393562

[50] Komatsu T, Sekiya T, Nagata K. 2013. DNA replication-dependent binding of CTCF plays a critical role in adenovirus genome functions. Sci Rep 3:2187. doi:10.1038/srep02187 23851926 PMC3711053

[51] Carson CT, Orazio NI, Lee DV, Suh J, Bekker-Jensen S, Araujo FD, Lakdawala SS, Lilley CE, Bartek J, Lukas J, Weitzman MD. 2009. Mislocalization of the MRN complex prevents ATR signaling during adenovirus infection. EMBO J 28:652–662. doi:10.1038/emboj.2009.15 19197236 PMC2666027

[52] Ching W, Koyuncu E, Singh S, Arbelo-Roman C, Hartl B, Kremmer E, Speiseder T, Meier C, Dobner T. 2013. A ubiquitin-specific protease possesses a decisive role for adenovirus replication and oncogene-mediated transformation. PLoS Pathog 9:e1003273. doi:10.1371/journal.ppat.1003273 23555268 PMC3610741

[53] Saha B, Parks RJ. 2019. Histone deacetylase inhibitor suberoylanilide hydroxamic acid suppresses human adenovirus gene expression and replication. J Virol 93:e00088-19. doi:10.1128/JVI.00088-19 30944181 PMC6613751

[54] Kelnhofer-Millevolte LE, Arnold EA, Nguyen DH, Avgousti DC. 2024. Controlling much? viral control of host chromatin dynamics. Annu Rev Virol 11:171–191. doi:10.1146/annurev-virology-100422-011616 38684115 PMC13244615

[55] Chen R, Kang R, Tang D. 2022. The mechanism of HMGB1 secretion and release. Exp Mol Med 54:91–102. doi:10.1038/s12276-022-00736-w 35217834 PMC8894452

[56] National Center for Biotechnology Information. PubChem pathway summary for pathway R-HSA-2559586, DNA damage/telomere stress induced senescence. Source: Reactome. PubChem. Available from: https://pubchem.ncbi.nlm.nih.gov/pathway/Reactome:R-HSA-2559586. Accessed 8 October 2024

[57] Pombo A, Ferreira J, Bridge E, Carmo-Fonseca M. 1994. Adenovirus replication and transcription sites are spatially separated in the nucleus of infected cells. EMBO J 13:5075–5085. doi:10.1002/j.1460-2075.1994.tb06837.x 7957073 PMC395454

[58] Maul GG, Jensen DE, Ishov AM, Herlyn M, Rauscher FJ. 1998. Nuclear redistribution of BRCA1 during viral infection. Cell Growth Differ 9:743–755. 9751118

[59] Pardo-Mateos A, Young CSH. 2004. Adenovirus IVa2 protein plays an important role in transcription from the major late promoter in vivo. Virology (Auckl) 327:50–59. doi:10.1016/j.virol.2004.06.011 15327897

[60] Avgousti DC, Herrmann C, Kulej K, Pancholi NJ, Sekulic N, Petrescu J, Molden RC, Blumenthal D, Paris AJ, Reyes ED, Ostapchuk P, Hearing P, Seeholzer SH, Worthen GS, Black BE, Garcia BA, Weitzman MD. 2016. A core viral protein binds host nucleosomes to sequester immune danger signals. Nature New Biol 535:173–177. doi:10.1038/nature18317 PMC4950998 27362237

[61] Mollica L, De Marchis F, Spitaleri A, Dallacosta C, Pennacchini D, Zamai M, Agresti A, Trisciuoglio L, Musco G, Bianchi ME. 2007. Glycyrrhizin binds to high-mobility group box 1 protein and inhibits its cytokine activities. Chem Biol 14:431–441. doi:10.1016/j.chembiol.2007.03.007 17462578

[62] Moisy D, Avilov SV, Jacob Y, Laoide BM, Ge X, Baudin F, Naffakh N, Jestin JL. 2012. HMGB1 protein binds to influenza virus nucleoprotein and promotes viral replication. J Virol 86:9122–9133. doi:10.1128/JVI.00789-12 22696656 PMC3416134

[63] Lichota J, Grasser KD. 2001. Differential chromatin association and nucleosome binding of the maize HMGA, HMGB, and SSRP1 proteins. Biochemistry 40:7860–7867. doi:10.1021/bi010548y 11425313

[64] Fan Z, Beresford PJ, Zhang D, Lieberman J. 2002. HMG2 interacts with the nucleosome assembly protein SET and is a target of the cytotoxic T-lymphocyte protease granzyme A. Mol Cell Biol 22:2810–2820. doi:10.1128/MCB.22.8.2810-2820.2002 11909973 PMC133744

[65] Dintilhac A, Bernués J. 2002. HMGB1 interacts with many apparently unrelated proteins by recognizing short amino acid sequences. J Biol Chem 277:7021–7028. doi:10.1074/jbc.M108417200 11748221

[66] Kugel S, Mostoslavsky R. 2014. Chromatin and beyond: the multitasking roles for SIRT6. Trends Biochem Sci 39:72–81. doi:10.1016/j.tibs.2013.12.002 24438746 PMC3912268

[67] Lam YW, Evans VC, Heesom KJ, Lamond AI, Matthews DA. 2010. Proteomics analysis of the nucleolus in adenovirus-infected cells. Mol Cell Proteom 9:117–130. doi:10.1074/mcp.M900338-MCP200 PMC2808258 19812395

[68] Genoveso MJ, Hisaoka M, Komatsu T, Wodrich H, Nagata K, Okuwaki M. 2020. Formation of adenovirus DNA replication compartments and viral DNA accumulation sites by host chromatin regulatory proteins including NPM1. FEBS J 287:205–217. doi:10.1111/febs.15027 31365788

[69] Dybas JM, Lum KK, Kulej K, Reyes ED, Lauman R, Charman M, Purman CE, Steinbock RT, Grams N, Price AM, Mendoza L, Garcia BA, Weitzman MD. 2021. Adenovirus remodeling of the host proteome and host factors associated with viral genomes. mSystems 6:e0046821. doi:10.1128/msystems.00468-21 PMC12338147 34463575

[70] Haruki H, Gyurcsik B, Okuwaki M, Nagata K. 2003. Ternary complex formation between DNA-adenovirus core protein VII and TAF-Ibeta/SET, an acidic molecular chaperone. FEBS Lett 555:521–527. doi:10.1016/s0014-5793(03)01336-x 14675767

[71] Haruki H, Okuwaki M, Miyagishi M, Taira K, Nagata K. 2006. Involvement of template-activating factor I/SET in transcription of adenovirus early genes as a positive-acting factor. J Virol 80:794–801. doi:10.1128/JVI.80.2.794-801.2006 16378981 PMC1346848

[72] Avgousti DC, Della Fera AN, Otter CJ, Herrmann C, Pancholi NJ, Weitzman MD. 2017. Adenovirus core protein VII downregulates the DNA damage response on the host genome. J Virol 91:e01089-17. doi:10.1128/JVI.01089-17 28794020 PMC5625504

[73] Del Moral-Morales A, Salgado-Albarrán M, Sánchez-Pérez Y, Wenke NK, Baumbach J, Soto-Reyes E. 2023. CTCF and its multi-partner network for chromatin regulation. Cells 12:1357. doi:10.3390/cells12101357 37408191 PMC10216408

[74] Huang H, Santoso N, Power D, Simpson S, Dieringer M, Miao H, Gurova K, Giam CZ, Elledge SJ, Zhu J. 2015. FACT proteins, SUPT16H and SSRP1, are transcriptional suppressors of HIV-1 and HTLV-1 that facilitate viral latency. J Biol Chem 290:27297–27310. doi:10.1074/jbc.M115.652339 26378236 PMC4646377

[75] Fox HL, Dembowski JA, DeLuca NA. 2017. A herpesviral immediate early protein promotes transcription elongation of viral transcripts. MBio 8:e00745-17. doi:10.1128/mBio.00745-17 28611249 PMC5472187

[76] Koyuncu E, Budayeva HG, Miteva YV, Ricci DP, Silhavy TJ, Shenk T, Cristea IM. 2014. Sirtuins are evolutionarily conserved viral restriction factors. MBio 5:e02249-14. doi:10.1128/mBio.02249-14 25516616 PMC4271551

[77] Ding X, Li S, Zhu L. 2021. Potential effects of HMGB1 on viral replication and virus infection-induced inflammatory responses: a promising therapeutic target for virus infection-induced inflammatory diseases. Cytokine Growth Factor Rev 62:54–61. doi:10.1016/j.cytogfr.2021.08.003 34503914

[78] Watt F, Molloy PL. 1988. High mobility group proteins 1 and 2 stimulate binding of a specific transcription factor to the adenovirus major late promoter. Nucleic Acids Res 16:1471–1486. doi:10.1093/nar/16.4.1471 2831501 PMC336328

[79] Lu B, Nakamura T, Inouye K, Li J, Tang Y, Lundbäck P, Valdes-Ferrer SI, Olofsson PS, Kalb T, Roth J, Zou Y, Erlandsson-Harris H, Yang H, Ting JP-Y, Wang H, Andersson U, Antoine DJ, Chavan SS, Hotamisligil GS, Tracey KJ. 2012. Novel role of PKR in inflammasome activation and HMGB1 release. Nature New Biol 488:670–674. doi:10.1038/nature11290 PMC4163918 22801494

[80] Darweesh M, Kamel W, Gavrilin MA, Akusjärvi G, Svensson C. 2019. Adenovirus VA RNAI blocks ASC oligomerization and inhibits NLRP3 inflammasome activation. Front Immunol 10:2791. doi:10.3389/fimmu.2019.02791 31849970 PMC6901988

[81] Tang Z, Zang N, Fu Y, Ye Z, Chen S, Mo S, Ren L, Liu E. 2018. HMGB1 mediates HAdV-7 infection-induced pulmonary inflammation in mice. Biochem Biophys Res Commun 501:1–8. doi:10.1016/j.bbrc.2018.03.145 29571731

[82] Groitl P, Dobner T. 2007. Construction of adenovirus type 5 early region 1 and 4 virus mutants. Methods Mol Med 130:29–39. doi:10.1385/1-59745-166-5:29 17401162

[83] Kosulin K, Lam E, Heim A, Dobner T, Rodríguez E. 2018. Broad-spectrum antiviral activity of the deubiquitinase inhibitor HBX against human adenoviruses. Antivir Ther 23:475–483. doi:10.3851/IMP3230 29557344

[84] Cox J, Mann M. 2008. MaxQuant enables high peptide identification rates, individualized p.p.b.-range mass accuracies and proteome-wide protein quantification. Nat Biotechnol 26:1367–1372. doi:10.1038/nbt.1511 19029910

[85] R Studio Core Team. 2020. RStudio: integrated development for R. RStudio, PBC. Boston, MA (USA). Available from: http://www.rstudio.com

[86] Maechler M, Rousseeuw P, Struyf A, Hubert M, Hornik K. 2023. Cluster: cluster analysis basics and extensions. Available from: https://CRAN.R-project.org/package=cluster

[87] Perez-Riverol Y, Bai J, Bandla C, García-Seisdedos D, Hewapathirana S, Kamatchinathan S, Kundu DJ, Prakash A, Frericks-Zipper A, Eisenacher M, Walzer M, Wang S, Brazma A, Vizcaíno JA. 2022. The PRIDE database resources in 2022: a hub for mass spectrometry-based proteomics evidences. Nucleic Acids Res 50:D543–D552. doi:10.1093/nar/gkab1038 34723319 PMC8728295

[88] Schindelin J, Arganda-Carreras I, Frise E, Kaynig V, Longair M, Pietzsch T, Preibisch S, Rueden C, Saalfeld S, Schmid B, Tinevez J-Y, White DJ, Hartenstein V, Eliceiri K, Tomancak P, Cardona A. 2012. Fiji: an open-source platform for biological-image analysis. Nat Methods 9:676–682. doi:10.1038/nmeth.2019 22743772 PMC3855844

[89] Ching W, Dobner T, Koyuncu E. 2012. The human adenovirus type 5 E1B 55-kilodalton protein is phosphorylated by protein kinase CK2. J Virol 86:2400–2415. doi:10.1128/JVI.06066-11 22190719 PMC3302271

[90] Koyuncu OO, Dobner T. 2009. Arginine methylation of human adenovirus type 5 L4 100-kilodalton protein is required for efficient virus production. J Virol 83:4778–4790. doi:10.1128/JVI.02493-08 19264777 PMC2682095

[91] Reich NC, Sarnow P, Duprey E, Levine AJ. 1983. Monoclonal antibodies which recognize native and denatured forms of the adenovirus DNA-binding protein. Virol (Auckl) 120:480–484. doi:10.1016/0042-6822(83)90274-x 6310869

