# Supplementary material for: The protein composition of human adenovirus replication compartments
Source: mBio. 2024 Nov 29;16(1):e02144-24. doi: 10.1128/mbio.02144-24 (PMC11708036; doi:10.1128/mbio.02144-24)
Supplement: Supplemental Figures — Fig. S1 to S9. [file mbio.02144-24-s0001.pdf]

Supplementary Information

The Protein Composition of Human Adenovirus Replication Compartments

by P. Hidalgo *et al*.

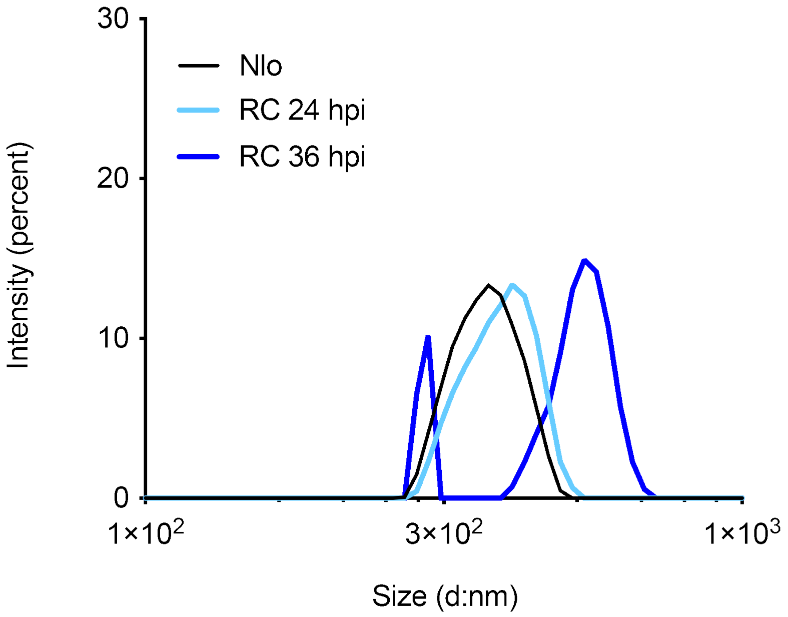

Fig. S1: DLS of RC-isolated fractions. The apparent hydrodynamic diameter was calculated using the Malvern Zetasizer Nano SP instrument at 4°C for each sample (nucleoli (Nlo/mock-infected RCs) or RC-enriched fractions at 24 or 36 hpi). The average intensity per diameter size in nm (d:nm) was plotted for each sample.

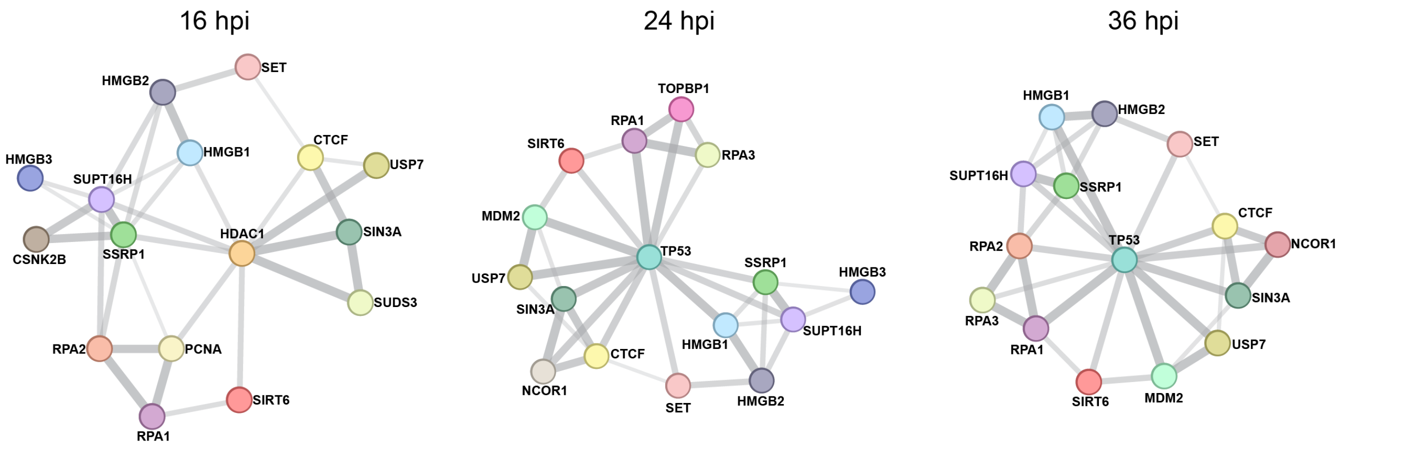

Fig. S2: Network analysis of RC-proteins using the STRING database. STRING interaction-network visualization of selected proteins previously reported to be in RCs and newly identified proteins of potential relevance for RCs, at 16, 24 and 36 hpi.

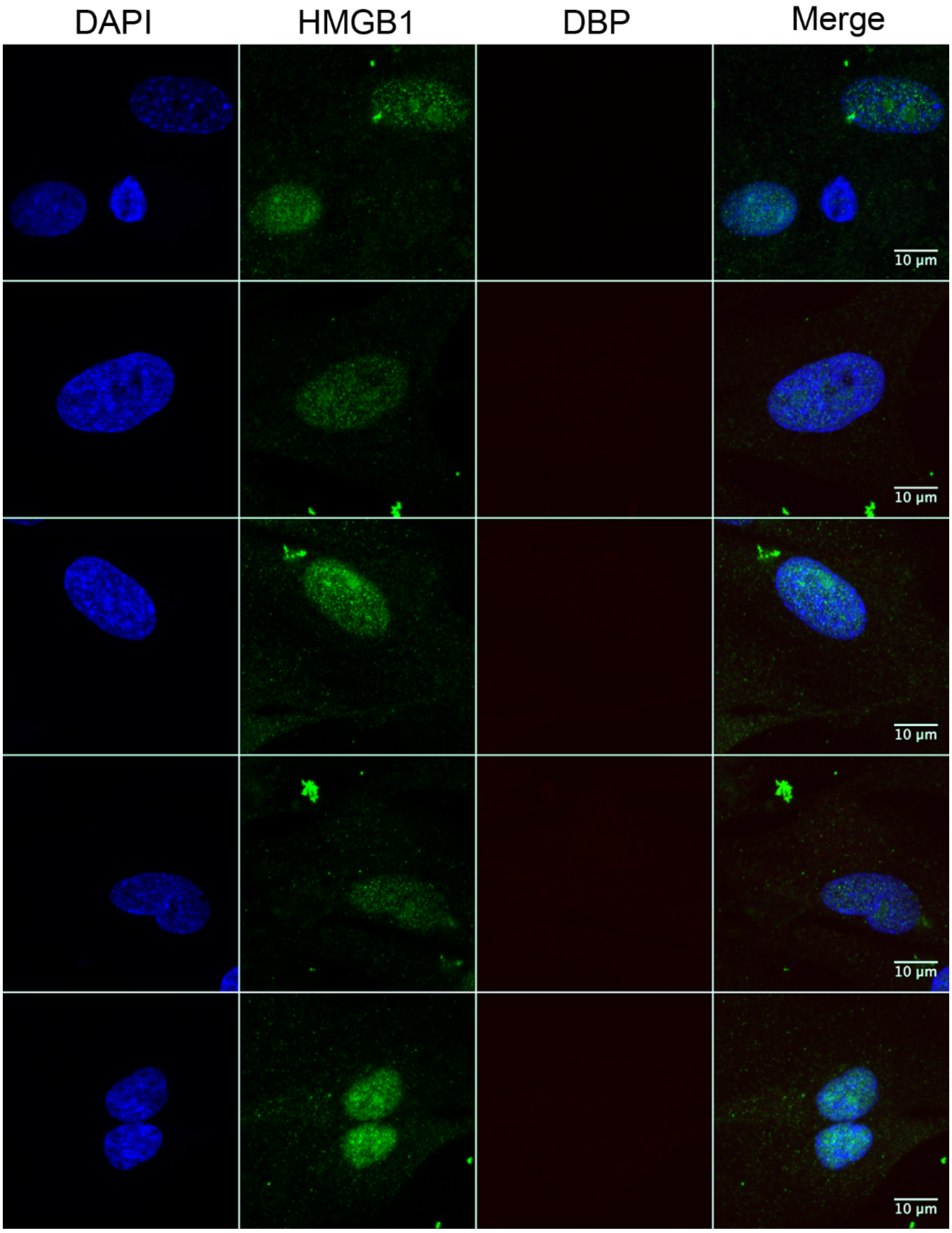

Fig. S3: HMGB1 localization in mock-infected cells. Mock-infected HFFs were fixed, stained for HMGB1 (green), DBP (red) and DNA (DAPI, blue) and visualized by confocal microscopy as z-stacks. The images are presented as maximum-intensity projections. The scale bars correspond to 10 µm.

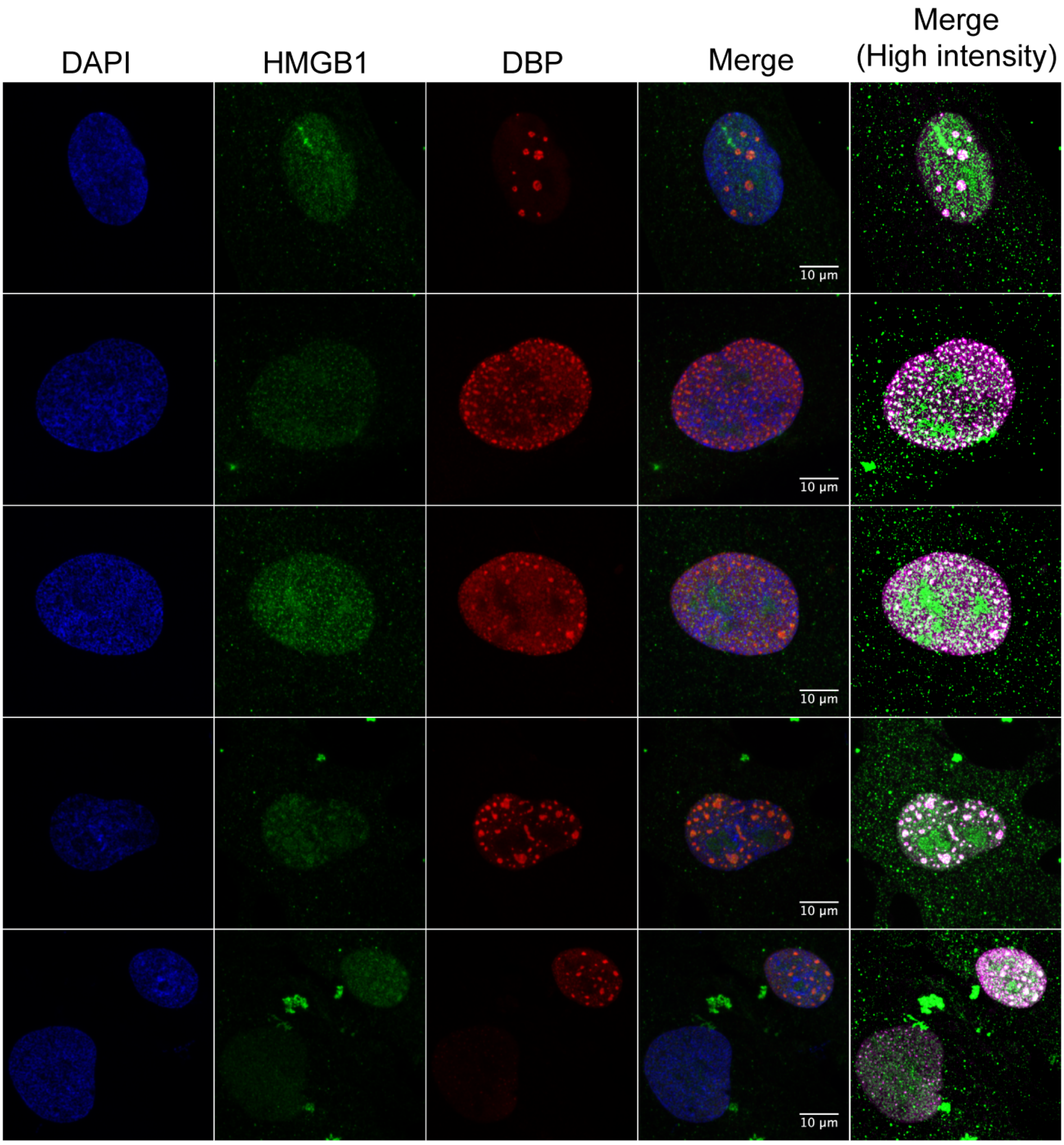

Fig. S4: HMGB1 localization at 16 hpi. HFFs infected with HAdV-C5 were fixed at 16 hpi, stained for HMGB1 (green), DBP (red) and DNA (DAPI, blue) and visualized by confocal microscopy as z-stacks. The images are presented as maximum-intensity projections. A high intensity column is included only with the purpose to show that, despite low levels of HMGB1 in some cells, it can be localized in RCs. This includes the overlap of DBP (magenta) and HMGB1 (green). The scale bars correspond to 10 µm.

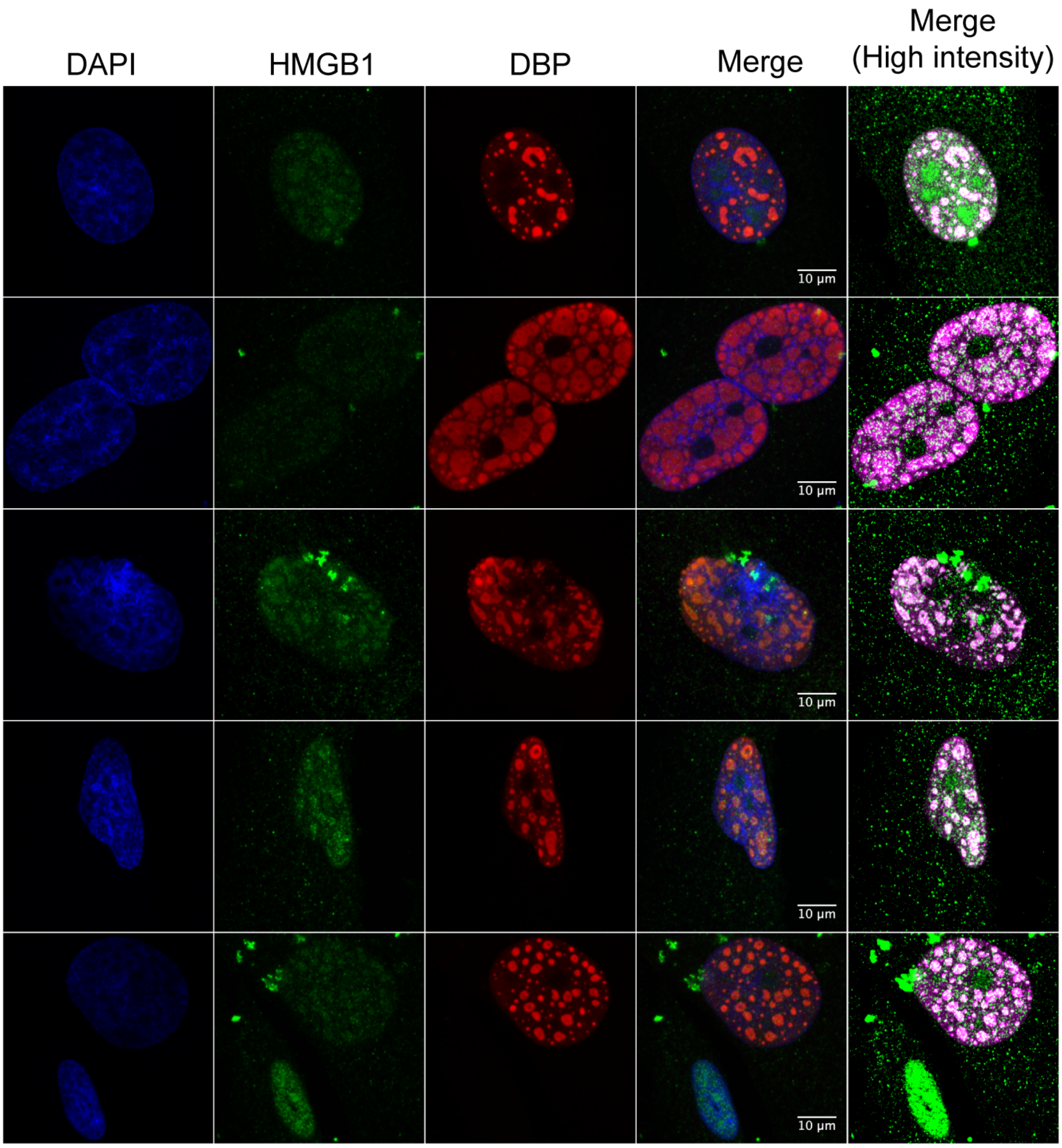

Fig. S5: HMGB1 localization at 24 hpi. HFFs infected with HAdV-C5 were fixed at 24 hpi, stained for HMGB1 (green), DBP (red) and DNA (DAPI, blue) and visualized by confocal microscopy as z-stacks. The images are presented as maximum-intensity projections. A high intensity column is included only with the purpose to show that, despite low levels of HMGB1 in some cells, it can be localized in RCs. This includes the overlap of DBP (magenta) and HMGB1 (green). The scale bars correspond to 10 µm.

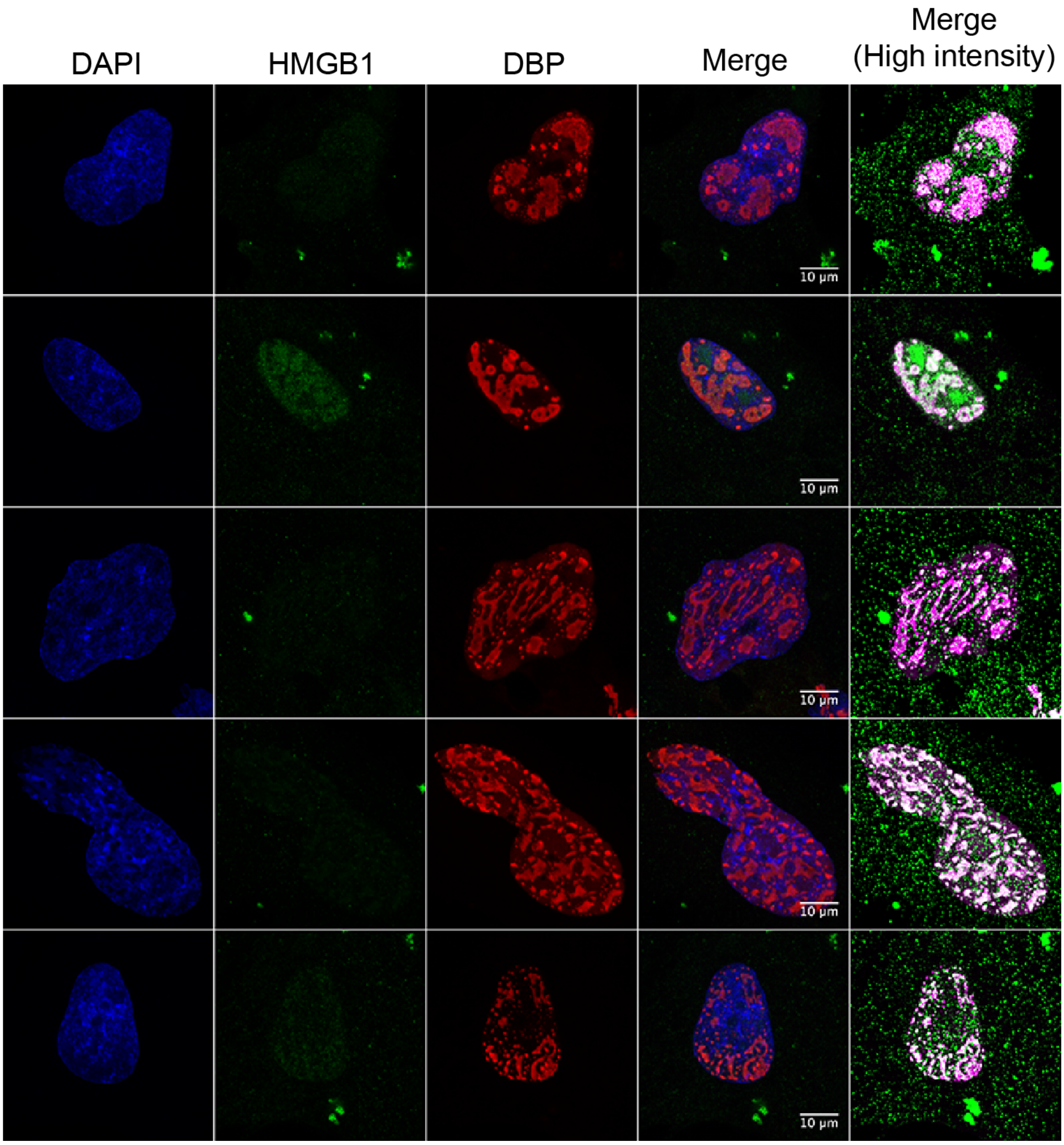

Fig. S6: HMGB1 localization at 36 hpi. HFFs infected with HAdV-C5 were fixed at 36 hpi, stained for HMGB1 (green), DBP (red) and DNA (DAPI, blue) and visualized by confocal microscopy as z-stacks. The images are presented as maximum-intensity projections. A high intensity column is included only with the purpose to show that, despite low levels of HMGB1 in some cells, it can be localized in RCs. This includes the overlap of DBP (magenta) and HMGB1 (green). The scale bars correspond to 10 µm.

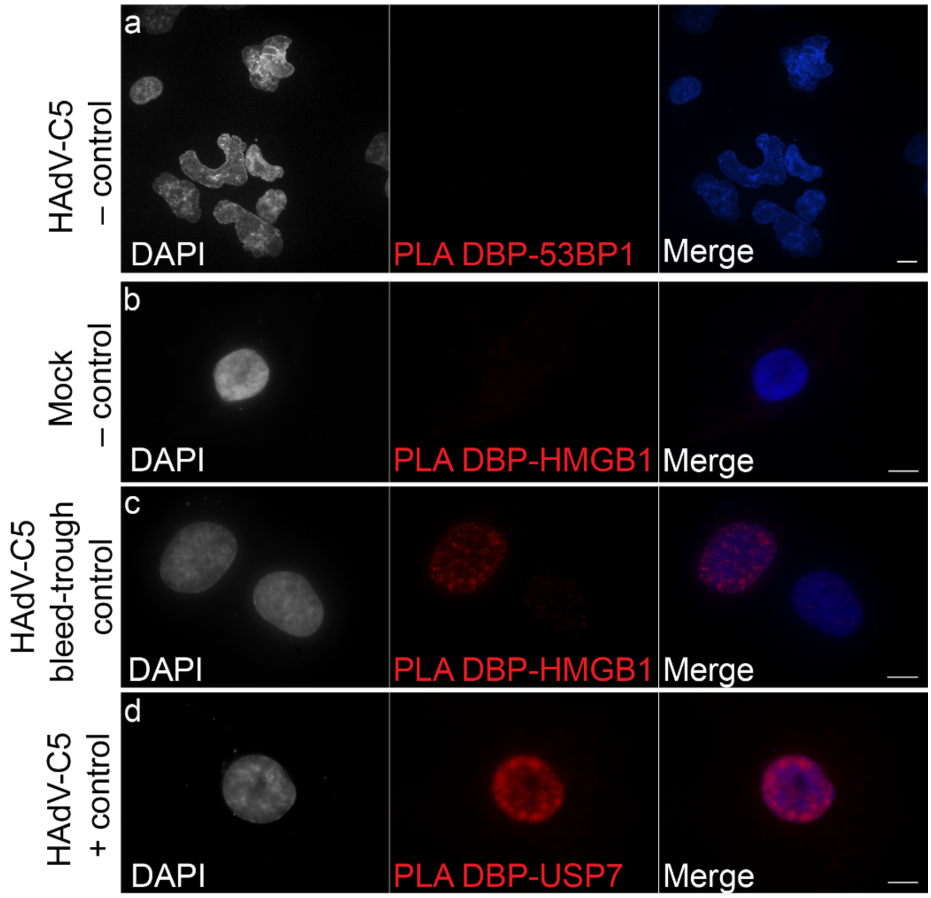

Fig. S7: PLA controls. PLAs were performed as described in the materials and methods section. The following controls for the PLAs were included: (a) As a negative control, HAdV-C5-infected A549 cells were subjected to PLA using the primary mAb against 53BP1 (a protein not interacting with DBP) and DBP; (b) An additional negative control included mock-infected A549 cells subjected to PLA using the primary mAb against HMGB1 and DBP; (c) To verify that the positive PLA-signal between DBP and HMGB1 was not due to a fluorescence bleed-through artefact, immunostaining of DBP was not included; (d) As a positive control, HAdV-C5-infected A549 cells were subjected to PLA using the primary mAb against USP7 (a protein known to bind to DBP) and DBP. All samples were visualized by confocal microscopy as z-stacks, and the images are presented as maximum-intensity projections. The scale bars correspond to 10 µm.

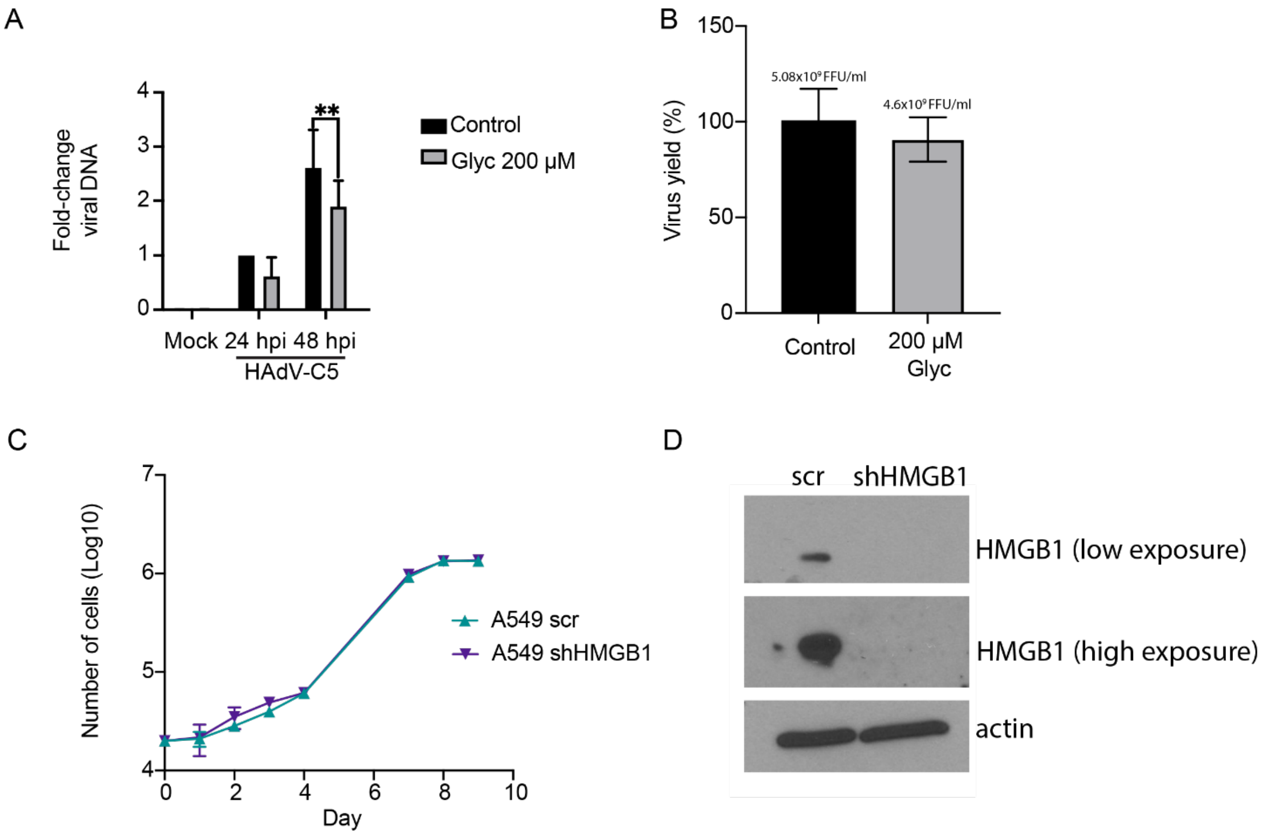

Fig. S8: Inhibition and knockdown of HMGB1. (A) HAdV-C5 DNA levels were measured by quantitative PCR at 24 and 48 hpi, in control cells or in cells treated with 200 μM Glyc. (B) Virus yield was determined by quantitative DBP immunofluorescence from control A549 cells or cells treated with 200 μM Glyc, infected with HAdV-C5 and harvested 48 hpi. (C) Cell growth curve for uninfected scr cells and cells that stably express shHMGB1 cells. The cells were harvested at the indicated days post seeding and the number of viable cells was determined. (D) Knockdown of HMGB1 was verified by immunoblotting in uninfected scr cells and cells that stably express shHMGB1. Actin was used as loading control. **p<0.01. For viral DNA data, multiple t tests with a two-stage step-up method of Benjamini, Krieger and Yekutieli False Discovery Rate (FDR) approach were used. For virus yield data, unpaired t tests were used.

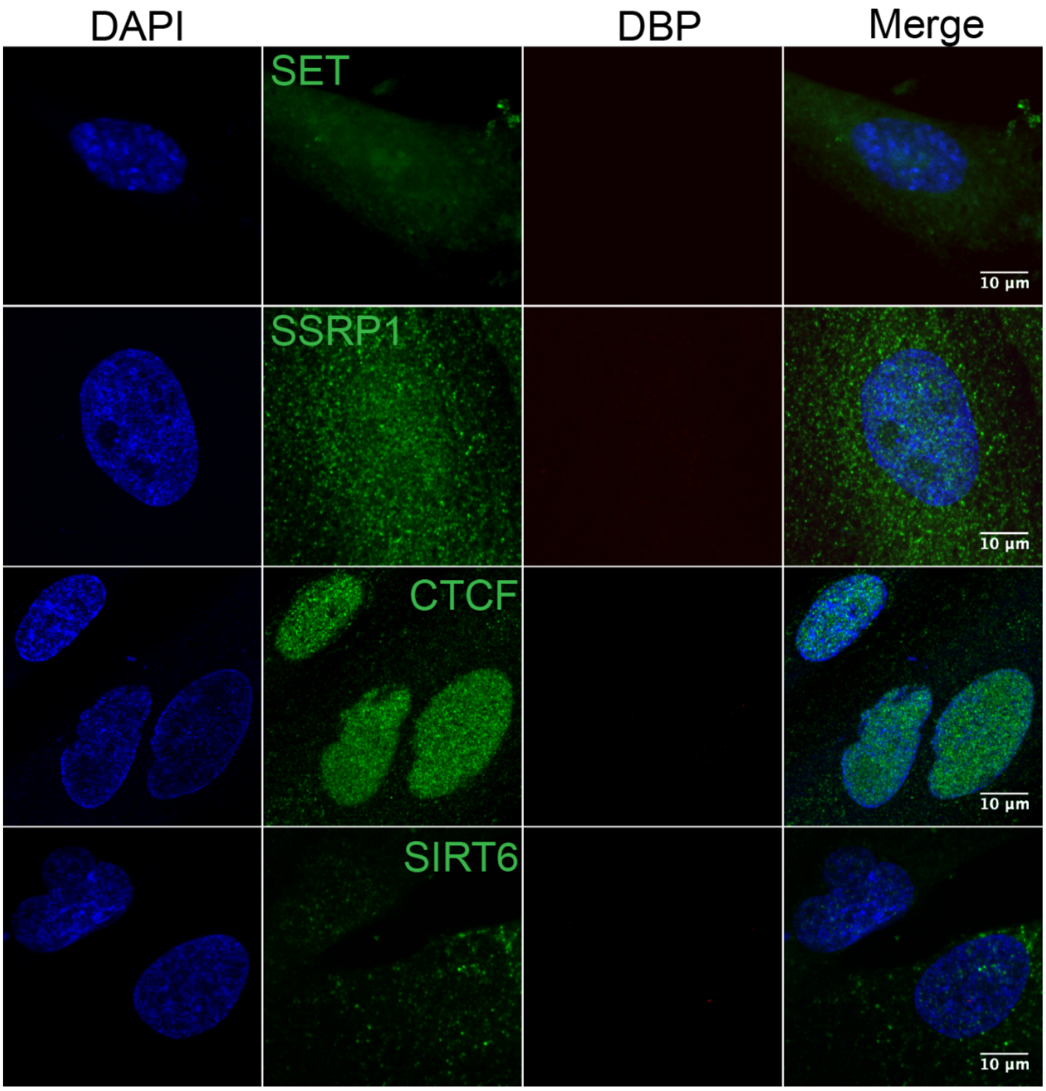

Fig. S9: Localization of proteins organizing chromatin architecture in mock-infected cells. Mock-infected A549 cells were fixed at 20 hpi. SET, SSRP1, CTCF or SIRT6 (green) were co-stained together with DBP (red) and DNA (DAPI, blue) and visualized by confocal microscopy as z-stacks. The images are presented as maximum-intensity projections. The scale bars correspond to 10 µm.
